# LFU-Net: LoRA-enhanced frequency-aware U-Net with recursive residual attention fusion for retinal segmentation

**DOI:** 10.1038/s41598-026-50642-8

**Published:** 2026-05-02

**Authors:** Zhongshi Wang, Xiaobing Chen, Zhanli Wang, Peng Shao, Yujie Luan, Yunxia Hu, Xiulan Kang, Xue Han, Zhifei Wang

**Affiliations:** 1 School of Mathematics and Information Sciences, Chaoyang Normal University, Chaoyang, 122000 China; 2 Department of Geriatrics, Chaoyang Central Hospital Affiliated to China Medical University, Chaoyang, 122000 China

**Keywords:** Retinal segmentation, LoRA-enhanced, Attention fusion, Frequency-aware, Computational biology and bioinformatics, Engineering, Mathematics and computing

## Abstract

Long-tail segmentation is a crucial challenge in computer vision, where most models prioritize common head classes over rare tail classes. This problem is particularly prominent in retinal vessel segmentation, as conventional approaches often struggle to overcome underrepresented faint vessels, noise-induced boundary ambiguity, and excessive parameters that prohibit portable deployment. To address these challenges, we introduce LFU-Net, a lightweight and clinically applicable method for long-tail retinal vessel segmentation. It integrates a three-component ensemble: a Frequency-Aware Encoder with a Multi-Branch Frequency Convolution block, which uses wavelet decomposition to suppress noise and retain details; Hierarchical frequency-token enhanced Low-Rank Adaptation, which efficiently enhances the representation of tail classes (faint vessels) with minimal parameters; and a Recursive Residual Attention Fusion module to ensure vascular topological continuity. Extensive experiments on four public benchmark datasets demonstrate that LFU-Net achieves competitive performance compared to recent relevant models. Its lightweight nature supports real-time inference on portable devices. Ablation studies confirm the improvement contribution of each core component, indicating its potential utility in early disease detection when clinical resources are limited.

## Introduction

Blindness and vision loss (BVL) are major public health concerns. Noncommunicable diseases (NCDs), such as cataracts, glaucoma, age-related macular degeneration, and diabetic retinopathy, are the leading causes of vision impairment globally^1^. Retinal vascular morphology information is of great significance for the treatment of many diseases^2^. For example, diabetic retinopathy (DR), a complication of diabetes, can lead to leakage and swelling of retinal vessels. Similarly, hypertensive retinopathy (HR) can result in the twisting or narrowing of retinal vessels. Therefore, analyzing retinal vessels in fundus images is an important approach to the early detection of various serious diseases. The integrity of vascular morphology, particularly that of faint vessels (capillaries and venules), directly correlates with disease staging. Hence, in the early diagnosis of some diseases, tail-like samples often carry disproportionate clinical importance^3^.

The segmentation of long-tail features of retinal vessels has emerged as a challenging task within the realm of computer vision. It is marked by a severe imbalance between the “head class” (frequent samples) and the “tail class”^4^. Models typically prioritize the optimization of the head class loss, which consequently leads to under - detection or false positives in the tail class. In recent years, the advent of classic frameworks such as U-Net^5^ and its variants has augmented vascular features via spatial attention or skip connections. However, this approach has also given rise to feature mismatch across scales. TransUNet^6^ models are capable of leveraging global attention to capture remote topological structures. Nevertheless, their substantial number of parameters renders them unsuitable for portable devices. Hence, it is an important research direction to develop an accurate retinal segmentation model that can be conveniently employed in clinical scenarios.

Taking advantage of frequency-domain feature decomposition’s ability to preserve the topology of long-tail samples and the potential of parameter-efficient tuning for lightweight adaptation, we developed LFU-Net, a lightweight framework tailored for long-tail vessel segmentation. Designed specifically for clinical portable retinal vessel detection, this framework addresses the limitations of existing methods in long-tail representation, cross-scale matching, noise-induced ambiguity, and portable deployment through three complementary blocks:

- Multi-Branch Frequency Convolution (**MBFC**) Block: Serving as the core of the Frequency-Aware Encoder (FAE), it employs Daubechies wavelet decomposition to separate high-frequency vessel edges from noise. It also integrates C/S-Attention to refine boundary details, forming a dual-mechanism design that resolves noise-induced ambiguity while preserving the topological characteristics of faint vessels.
- Frequency token LoRA (**FreqTokenLoRA**) Block: It targetedly enhances the feature representation of tail classes, while hierarchical LoRA^7^ fixed-rank helps reduce the number of parameters to enable efficient and portable deployment. This design achieves targeted enhancement of faint vessel features without introducing redundant computations.
- Recursive Residual Attention Fusion (**RRAF**) Block: It iteratively refines features via QKV and channel-spatial attention alongside residual connections. It specifically weights tail-class features, models cross-scale dependencies and preserves the topology of faint vessels, effectively mitigating the continuity loss of weak vessel structures during feature fusion.

## Related work

**Fig. 1:**
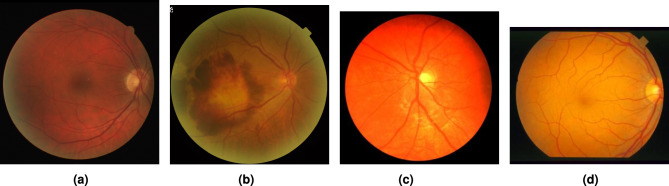
Imaging heterogeneity across the four benchmark datasets.

Retinal vessel segmentation has undergone two primary stages. In the initial stage, it primarily relied on image processing and traditional machine learning algorithms, which involved filtering, thresholding, and morphological operations, and utilized the geometric and grayscale features of blood vessels for segmentation. For instance, Gwevu et al.^8^ used normalized Gabor filters and automatic thresholding, Mapayi et al.^9^ combined phase congruency with CLAHE, and Xu et al.^10^ proposed an OTSU threshold + morphological segmentation strategy. Nevertheless, despite the high computational efficiency of these traditional methods, they fail to account for the inherent imaging variability introduced by diverse clinical scenarios (e.g., variable illumination) and heterogeneous imaging devices in practical clinical settings. As a result, they did not fundamentally resolve the problems of faint vessels integrity loss^11^ and poor generalization^12^ (Fig. 1).

With the continuous upgrading of the algorithm based on the Unet^5^ architecture of deep learning, the effect of retinal vessel segmentation has been significantly improved. UNet++^13^ introduced a nested UNet architecture that enhanced segmentation accuracy. Wang et al.^14^ proposed a dual-coded U-Net (DEU-Net), which improves the ability of the network to segment retinal blood vessels pixel-to-pixel. Zhang et al.^15^ presented an attention guided network (AG-Net), which designs a filter named the attention guided filter to obtain improved segmentation results for fundus images. Some scholars have introduced wavelet transform^16,17^ to supplement frequency domain information in order to solve the problem of breakage or disappearance of blood vessel tail ends. Although good results have been achieved, these advances have not fully solved the long-tail challenge: existing methods still have insufficient performance in capillary segmentation, or exchange accuracy at the cost of efficiency^18^,Table 1.

**Table 1:** The key contributions of each method.

Methods	Contribution	Limitation
Gabor filters^8^	Normalized gabor filters and automatic thresholding	Not fundamentally resolve the problems of faint vessel integrity loss and poor generalization.
CLAHE^9^	Global thresholding techniques	
OTSU threshold^10^	Morphological segmentation	
UNet^5^	Encoder-decoder structure; Skip connection	Still have insufficient performance in capillary segmentation, or exchange accuracy at the cost of efficiency.
UNet++^13^	Nested UNet structure	
DEU-Net^14^	Dual encoding network	
AG-Net^15^	Attention guided network	
LSW-net^16^	Wavelet transform	
LFA-Net^19^	LiteFusion attention focus on small vessel segmentation	
Dense U-Net^20^	Generate high-quality synthetic capillaries	

The lightweight model LFA-Net^19^ adopts the attention mechanism to cut down on parameters, making it well-suited for mobile devices. However, its capacity to capture multi-scale features is compromised, leading to failure to detect small blood vessels. In contrast, ensemble networks typically integrate high-precision models such as Pyramid Vision Transformer (PVT), FCN-Transformer^21^, and Dense U-Net^20^ enhanced with GAN. These networks come with substantial computational overhead, which limits their use in clinical real-time applications.

In 2022, Edward et al.^22^ proposed a Low-Rank Adaptation (LoRA) method to resolve the storage and deployment issues encountered during the full fine-tuning of large pre-trained language models. They used LoRA to freeze the weights of the pre-trained model while updating it by injecting trainable low-rank factorization matrices into each layer of the Transformer architecture. This approach quickly gained wide attention from researchers in the field of medical image segmentation. In 2024, Mahla et al.^23^ put forward MedSAGa, which performs medical image segmentation through Gradient Low-Rank Projection to enhance clinical usability. In 2025, Ghassen Baklouti et al.^24^ emphasized that organs differ in morphological complexity, requiring flexible rank selection, and they adopted Regularized Low-Rank Adaptation for Few-Shot Organ Segmentation. We find that although vanilla LoRA is more parameter-efficient than Full Fine-Tuning (FFT), with 900$\times$ fewer training parameters, it still lags behind in performance. Fan et al.^25^ further confirmed that medical small-target segmentation needs to dynamically adjust the modeling dimension based on target scale and texture sparsity. From the above analysis, we can deduce specific requirements for rank selection in retinal vessel segmentation models. For shallow layers, which cover both head-class thick vessels and tail-class faint vessels in high-resolution regions, a small rank is necessary. This small rank helps preserve the strong edges of thick vessels and the basic contours of faint vessels simultaneously while avoiding parameter redundancy. For deep layers, which target tail-class faint vessels and the complex topology of thin vessels, a larger rank is required. This larger rank is used to model the sparse and intricate topological patterns of retinal vessels.

## Methods

This section elaborates on the architectural design, mathematical formulations, and implementation details of our proposed LFU-Net (LoRA-Enhanced Frequency-Aware U-Net with Recursive Residual Attention Fusion), a lightweight framework tailored to long-tail retinal vessel segmentation. Conventional methods face three intertwined challenges. These include underrepresented faint vessels, noise-induced boundary ambiguity, and excessive parameters that limit portable deployment. Simple solutions or single modules cannot address these comprehensively. Standalone frequency decomposition effectively suppresses noise but lacks the capacity to enhance subtle vascular structures. Single attention fusion fails to tackle the underlying long-tail class imbalance in vessel segmentation. While independent LoRA adaptation reduces the model’s parameter count, it neither mitigates noise-induced degradation nor preserves vascular structural continuity. Specifically, LFU-Net directly tackles three domain-specific issues: (1) under-representation of faint vessels caused by severe class imbalance; (2) boundary ambiguity arising from medical image noise, which is efficiently mitigated through frequency-domain analysis; (3) lightweight parameterization that enables the model to be deployed in portable clinical devices.

### Network architecture

**Fig. 2:**
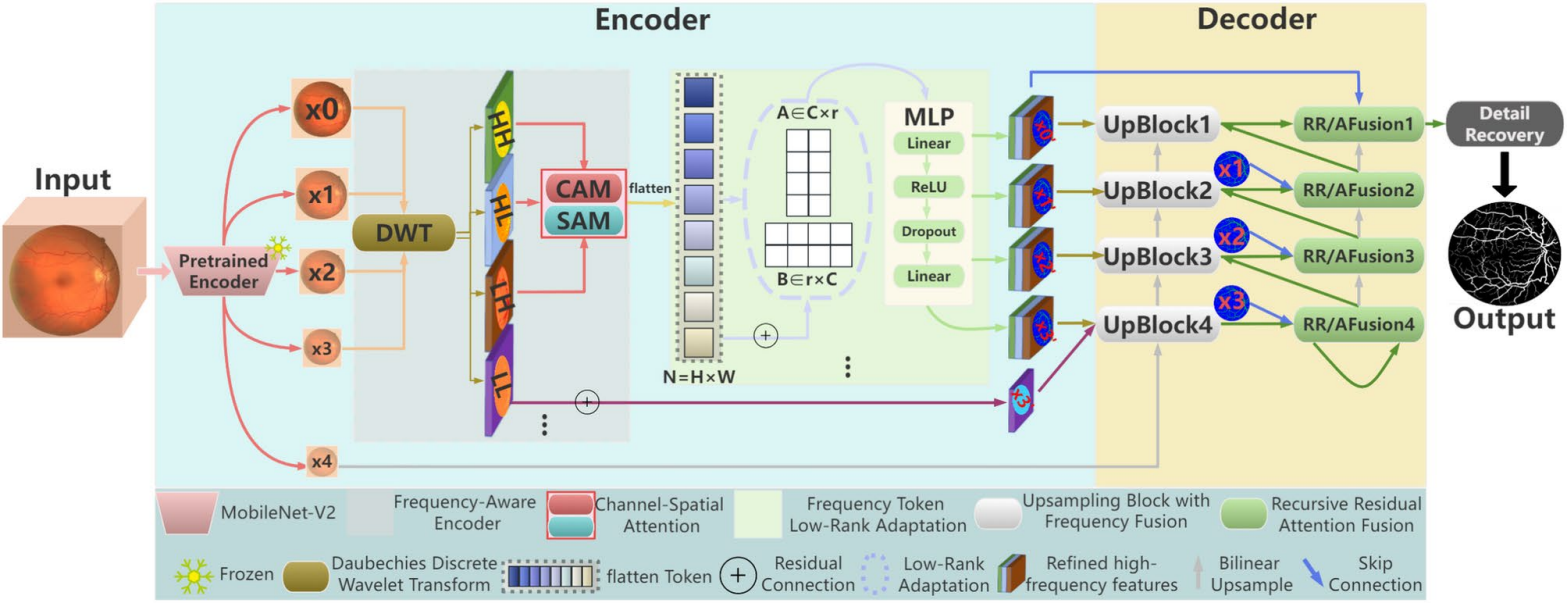
The overall structure of LFU-Net.

The proposed LFU-Net adopts a hybrid encoder-decoder architecture that combines the strengths of different network types. It builds upon pretrained feature extraction and incorporates frequency-domain analysis, attention mechanisms, and Low-Rank Adaptation (LoRA) to enhance the feature representation of faint vessels while maintaining robustness to class imbalance (Fig. 2).

The architecture of LFU-Net is built upon a pretrained MobileNetV2 backbone^26^. Specifically, The FreqTokenLoRA module is applied to the hierarchical feature outputs of the pretrained encoder, extracting finer hierarchical spatial features through low-rank adaptation. They operate on the encoder’s output features, facilitating the extraction of refined hierarchical spatial representations without full backbone fine-tuning. This strategy effectively balances model adaptability and parameter efficiency. Additionally, the Daubechies DWT Module decomposes features via discrete wavelet transform to suppress background noise and preserve faint vessel boundaries. The C/S-Attention Modules refine feature representations by selectively emphasizing vessel regions, particularly faint vessels, while suppressing non-vessel interference to enhance the representation of tail-class structures. As the core module embedded in the skip-connection bridge, Recursive Residual Attention Fusion (RR/A Fusion) integrates cross-scale features along the skip connection to prioritize faint vessels and maintain vascular topological continuity. Finally, the Detail Recovery Block at the output stage fuses spatial and frequency-enhanced details to amplify the subtlety of faint vessels, ensuring accurate segmentation of faint vessels. For an input retinal image $\mathcal {I} \in \mathbb {R}^{H \times W \times 3}$ (resized to 480$\times$480 to balance resolution and computational efficiency), the framework’s signal flow is formally defined as (Table 2):

**Table 2:** Signal Flow Formulation.

Step	Mathematical Expression	Description
1	$\mathcal {I}_{\text {norm}} = \text {Normalize}(\mathcal {I})$	Preprocessing module
2	$X_0, X_1, X_2, X_3, X_4 = \text {PretrainedEncoder}(\mathcal {I}_{\text {norm}})$	Multi-scale feature extraction
3	$F_0, F_1, F_2, F_3 = \text {FAE}(X_0, X_1, X_2, X_3)$	Frequency-Aware Encoder
4	$X_0', X_1', X_2', X_3' = \text {LoRA}(F_0), \dots , \text {LoRA}(F_3)$	Tail-class feature enhancement
5	$D_4 = \text {Conv}_{1\times 1}(X_4)$	Deepest encoder initialization
6	$D_i = \text {RRAF}(D_{i+1}, X_i', S_i / \mathcal {M}_{\text {field}}),\; (i = 3, 2, 1, 0)$	Recursive skip fusion
7	$\mathrm {\hat{Y}=Softmax(DetailRecovery(D_0))}$	Binary segmentation logits

Where:

$$\begin{aligned} X_i&:X_i \in \mathbb {R}^{B \times C_i \times H_i \times W_i} , \text {Encoder features } \bigl (B = \text {batch size},\; C_i \in \{64, 128, 256, 512, 1024\},\; H_i = \frac{480}{2^i},\; W_i = \frac{480}{2^i}\bigr ); \\ \boldsymbol{F}_i&: \text {Frequency-refined features (i.e., the output of the Frequency-Aware Encoder);} \\ \boldsymbol{D}_i&: \text {Decoder features (i.e., the output of the LoRA-enhanced decoder);} \\ \boldsymbol{S}_i&: \text {High-resolution feature maps from the encoder;} \\ \mathcal {M}_{\text {field}}&:\mathcal {M}_{\text {field}}\in \mathbb {R}^{B \times 1 \times 480 \times 480} , \text {Valid field}_\text {mask;} \\ \boldsymbol{\hat{Y}}&: \text {Final output.} \end{aligned}$$

### Pretrained encoder

The pretrained encoder extracts multi-scale spatial features, while the layer-wise LoRA adapter enhances faint vessel features. Only minimal parameter updates are required for model adaptation, avoiding full fine-tuning. In retinal imaging, tail-class features are sparse, making full fine-tuning prone to overfitting and fixed-rank LoRA^27^ ineffective due to layer differences. This paper proposes an hierarchical fixed-rank LoRA that adjusts the rank based on feature sparsity, improving both parameter efficiency and tail-class representation.

#### Backbone initialization

The pretrained encoder is designed to strike a balance between general feature transfer and retinal-specific adaptation. It adopts a MobileNetV2 backbone^26^ (13.5M parameters) pretrained on ImageNet-1K^28^, where early layers (indices 0–99) are fixed to preserve universal low-level features such as edge and gradient cues, which can be effectively generalized to retinal vessel structures. In contrast, later bottleneck layers (indices 101–155) are fine-tuned to capture domain-specific patterns including vessel curvature and optic disc texture.A multi-scale feature extraction unit is utilized^29^, which derives four hierarchical feature maps from selected bottleneck layers of the backbone. These maps cover diverse spatial resolutions and channel dimensions that correspond to the structural hierarchy of retinal vessels. To ensure seamless integration with downstream modules, specifically the Frequency-Aware Encoder (FAE)^30^ and LoRA-based adapters^22^, we employ channel alignment convolutions (denoted as conv_x0–x3). These convolutions standardize the feature channel dimensions to 64, 128, 256, and 512 respectively, and this standardization eliminates dimensional inconsistencies during feature fusion.

Finally, layer-wise FreqTokenLoRA injection integrates hierarchical fixed ranks tailored to the sparsity of retinal vessel features^31^. Umirzakova et al.^32^ demonstrated that fixed hierarchical modules outperform adaptive counterparts in MRI-based lesion classification. Medical images have inherent structural regularity, and adaptive modules tend to overfit to this regularity. Retinal vessel features exhibit fixed hierarchical sparsity, allowing fixed ranks to closely match this inherent structure. In contrast, adaptive rank LoRA methods dynamically adjust based on feature distribution but require additional modules to predict and adjust the rank, which increases parameters and inference time. Our fixed-rank design effectively avoids such redundancy. Rank r=8 is provided for shallow features $\left( X_0\text {: }120 \times 120, X_1\text {: }60 \times 60\right)$ aimed at head-class thick vessels with simple topologies, while r=16 is assigned to deep features $\left( X_2\text {: }30 \times 30, X_3\text {: }15 \times 15\right)$targeting tail-class faint vessels with sparse features and complex topologies. This design effectively boosts the representation of tail-class vessels without introducing extra parameters, while safeguarding the consistency of dominant-class features.

#### Multi-scale feature extraction

The Pretrained Encoder takes advantage of predefined bottleneck layers in MobileNetV2 at indices 3, 6, 13, and 17, corresponding to distinct levels of the feature hierarchy.Shallow layers (indices 3, 6) are more focused on acquiring high-resolution edge-sensitive features for thick vessels. Deeper layers (indices 13, 17) store low-resolution semantic representations associated with faint vessel structures.High-resolution feature maps (stride=4) preserve fine-grained spatial details critical for detecting small capillaries. In contrast, the 15$\times$15 low-resolution maps (stride=32) gather global contextual information, supporting robust thick vessel segmentation. As spatial resolution reduces, channel dimensions increase in turn from 64 to 128, 256, and 512.This allocates greater representational capacity to context-rich, low-resolution features.This architectural design lays an outstanding preliminary foundation for subsequent frequency-aware cross-scale fusion. It facilitates effective integration of hierarchical features to optimize tail-class vessel detection (Table 3).

**Table 3:** Multi-scale features extracted by the pretrained encoder.

Feature map	Extract layer indices	Stride	Spatial size	Raw channels (MobileNetV2)	Aligned Channels	Lora rank
$X_0$	3	$2^2$	$120 \times 120$	24	64	8
$X_1$	6	$2^3$	$60 \times 60$	32	128	8
$X_2$	13	$2^4$	$30 \times 30$	96	256	16
$X_3$	17	$2^5$	$15 \times 15$	320	512	16
$X_4$	Final layer	$2^6$	$7 \times 7$	1280	1024	–

### Frequency-aware encoder

The Frequency-aware Encoder (FAE) overcomes the long-tail challenge by separating feature maps into spatial and frequency domains. It includes two key components, a db4 wavelet decomposition module and a Multi-Branch Frequency Convolution (MBFC) block, connected via an adaptive frequency fusion mechanism.

#### Wavelet decomposition

The Daubechies Discrete Wavelet Transform (DWT) module serves as the frequency decomposition core of the Frequency-Aware Encoder (FAE). It aims to extract multi-scale frequency components, enabling targeted handling of fine vessel details (high-frequency) and thick vessel contours (low-frequency) in retinal images.The wavelet decomposition unit adopts fixed db4 wavelet kernels that are non-trainable, performing wavelet decomposition on each LoRA-adapted feature $X_i'$ into four sub-bands. This prevents overfitting to optic disc noise. The decomposition process is formally defined as follows:

1 $$\begin{aligned} \text {LL}_i, \text {LH}_i, \text {HL}_i, \text {HH}_i = \text {DWT}(X_i') \end{aligned}$$

Where:

$$\begin{aligned} \text {LL}_i&:\text {LL}_i \in \mathbb {R}^{B \times C \times H/2 \times W/2}, \text {Captures global structures}; \\ \text {LH}_i&: \text {Captures horizontal faint vessel edges}; \\ \text {HL}_i&: \text {Captures vertical faint vessel edges}; \\ \text {HH}_i&: \text {Improves the detection capability of faint vessels in all directions}. \end{aligned}$$

#### Multi-branch frequency convolution

**Fig. 3:**
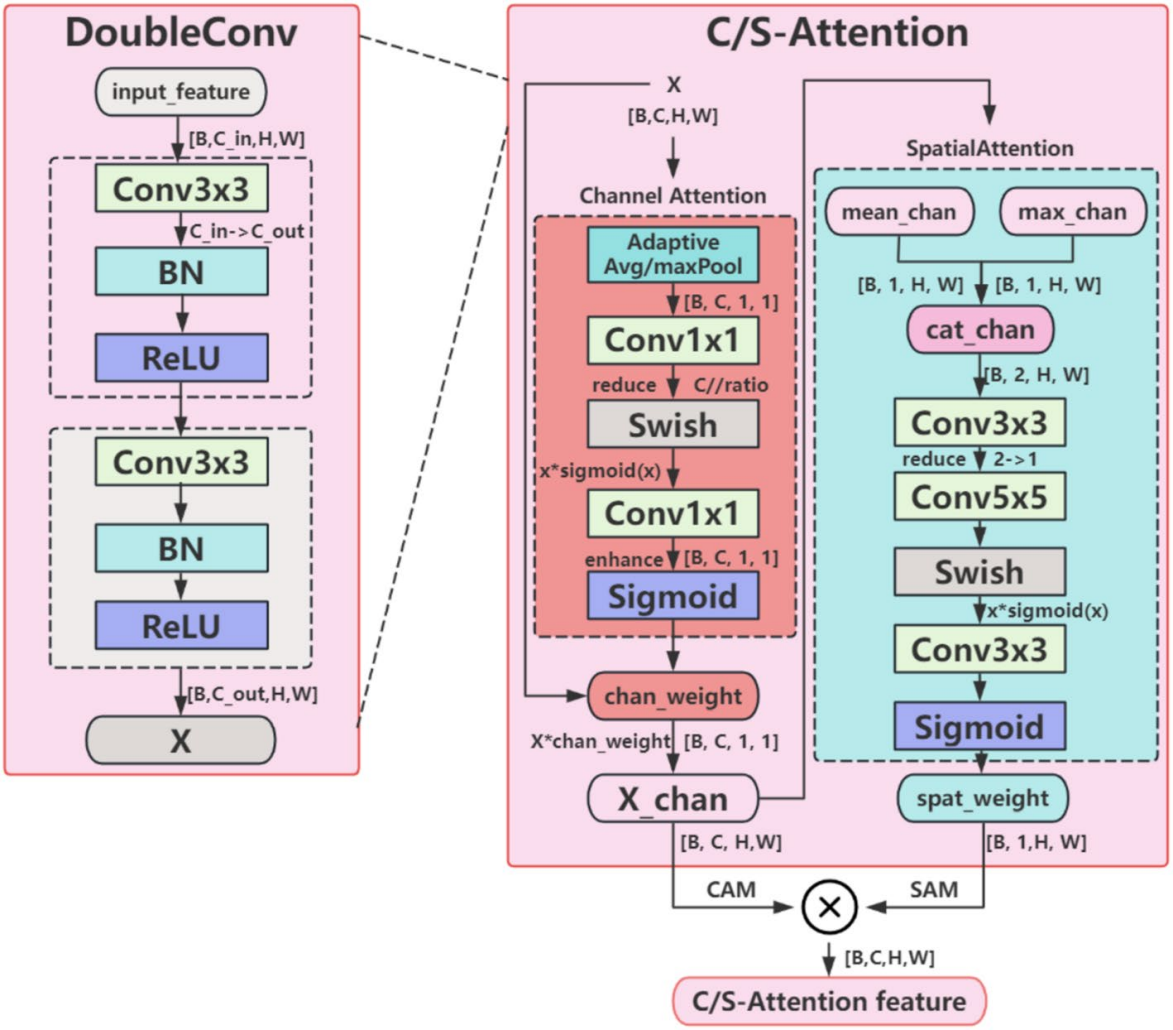
The architecture of the channel-spatial attention (C/S-Attention) unit.

The Multi-Branch Frequency Convolution (MBFC) Block tackles the limitations of fixed-frequency approaches^30^. It clearly differentiates high-frequency faint vessel cues from high-frequency noise, such as optic disc artifacts. Furthermore, it combines multi-scale frequency subbands with an attention mechanism^33^ to sharpen fine vessel structures, including capillary branches and low-contrast boundaries. It simultaneously suppresses irrelevant background interference, which improves the precision of retinal vessel segmentation (Fig. 3).

The C/S-Attention unit prioritizes channels and spatial regions related to faint vessels, refining the high-frequency sub-bands $HF_i$ . The Channel Attention Mechanism performs global average pooling (GAP) and channel dimensionality reduction to emphasize channels sensitive to faint vessels.

2 $$\begin{aligned} \alpha _c = \text {Sigmoid}\left( W_2 \cdot \text {Swish}\left( W_1 \cdot \text {GAP}(\text {HF}_i)\right) \right) \end{aligned}$$

Where:

$$\begin{aligned} \text {HF}_i&: \text {HF}_i \in \{\text {HH}_i, \text {LH}_i, \text {HL}_i\},\; \text {high-frequency features}; \\ W_1&: W_1 \in \mathbb {R}^{C \times C/4}; \\ W_2&: W_2 \in \mathbb {R}^{C/4 \times C},\; \text {channel reduction ratio=4}. \end{aligned}$$

The Spatial Attention Mechanism first processes the channel-refined feature map. It concatenates the mean and maximum pooling features of this map along the channel dimension. A spatial weight map is then obtained via a convolutional layer followed by sigmoid activation. This mechanism highlights spatial regions densely populated with faint vessels, utilizing global max pooling (GMP) and 3$\times$3 convolutions to suppress background noise.

3 $$\begin{aligned} \alpha _s = \text {Sigmoid}\left( \text {Conv}_{3\times 3}\left( \text {Cat}\left( \text {GAP}(\text {HF}_i \odot \alpha _c), \text {GMP}(\text {HF}_i \odot \alpha _c)\right) \right) \right) \end{aligned}$$

Dynamically adjusts the balance of high-frequency sub-bands ($\text {HH}_i, \text {LH}_i, \text {HL}_i$) via learnable weights that add up to one, preventing excessive weighting of any single component.

4 $$\begin{aligned}&\alpha _{\text {HH}}, \alpha _{\text {LH}}, \alpha _{\text {HL}} = \text {Sigmoid}\left( \text {Conv}_{1\times 1}\left( \text {Cat}\left( \text {HH}_i, \text {LH}_i, \text {HL}_i\right) \right) \right) \end{aligned}$$

5 $$\begin{aligned}&\text {HF}_{\text {refined},i} = \alpha _{\text {HH}} \cdot (\text {HH}_i \odot \alpha _c^{\text {HH}} \odot \alpha _s^{\text {HH}}) + \alpha _{\text {LH}} \cdot (\text {LH}_i \odot \alpha _c^{\text {LH}} \odot \alpha _s^{\text {LH}}) + \alpha _{\text {HL}} \cdot (\text {HL}_i \odot \alpha _c^{\text {HL}} \odot \alpha _s^{\text {HL}}) \end{aligned}$$

Refined high-frequency features ($\text {HF}_{\text {refined},i}$) are fused with low-frequency features $\text {LL}_i$ so that both faint vessel edges (tail-class) and global structure (head-class) are preserved. A $3\times 3$ convolution aligns their channel dimensions of $\text {HF}_{\text {refined},i}$ and $\text {LL}_i$ (output channels=*C*), ensuring no loss of tail-class information.

6 $$\begin{aligned} \text {F}_i = \text {LL}_i + \text {Conv}_{3\times 3}(\text {HF}_{\text {refined},i}) \end{aligned}$$

### Layer-wise LoRA for tail-class adaptation

**Fig. 4:**
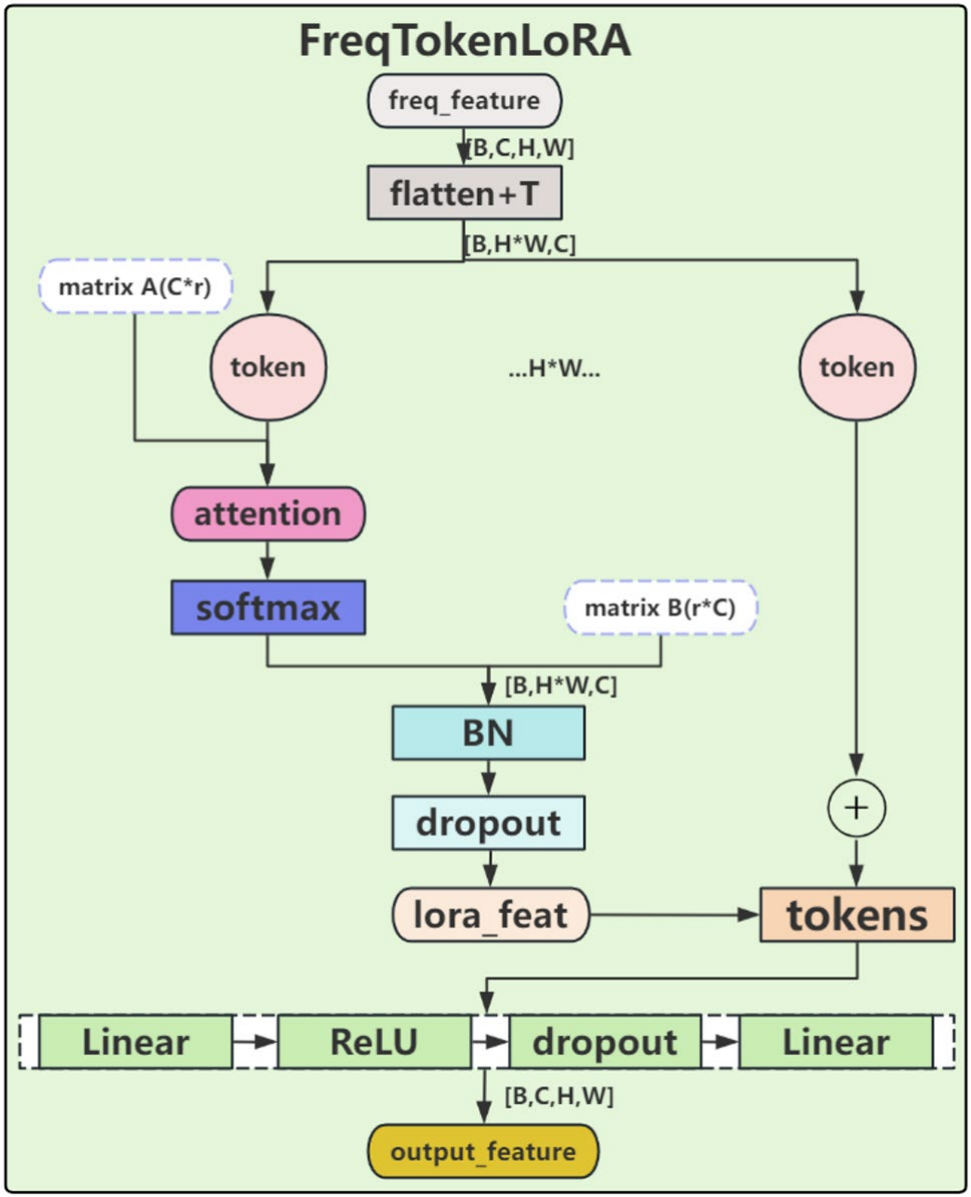
The architecture of the FreqTokenLoRA module.

Figure 4 shows the detailed structure of Layer-Wise LoRA for Tail-Class Adaptation (FreqTokenLoRA). It is proposed to introduce low-rank adaptation into feature transformation layers, adapting and boosting frequency-domain features with lower computational cost to focus on faint vessel (tail-class) representation in retinal images.

The FreqTokenLoRA module realizes core synergy by directionally integrating frequency-specific features and low-rank adaptation. Its input comes from high-frequency^34^ components (HL, LH, HH sub-bands) of the FAE module, which encapsulate detailed vascular topological information. Through LoRA’s low-rank matrix A, the module projects features into a low-dimensional subspace, selectively retaining discriminative fine vascular patterns such as capillary branching angles and diameter gradients. It simultaneously suppresses high-frequency noise like optic disc artifacts during this process. During reconstruction via matrix B, only low-rank updates related to tail-class vessels are preserved, minimizing interference with head-class vessel representations. This forms a closed-loop interaction with high-frequency detail extraction, whereby FreqTokenLoRA boosts the feature discriminability of these representations.

The layer-wise LoRA adapter is injected into $X_0$–$X_3$ to boost faint vessel features with minimal parameter overhead. LoRA-enhanced features ($X_0'$–$X_3'$) are fused with original aligned features via residual connections, preserving head-class (thick vessel) information while amplifying tail-class (faint vessel) signals. It adopts hierarchical fixed ranks ($r=8$ for shallow layers $X_0/X_1$, $r=16$ for deep layers $X_2/X_3$), aligned with retinal vessel feature sparsity. Shallow layers capture thick vessels with dense feature distributions, where a small rank avoids redundant parameterization. In contrast, deep layers target faint vessels with sparse topological patterns, requiring a higher rank for sufficient modeling and underfitting prevention. A pair of low-rank matrices (*A* and *B*) realizes task-specific adjustments: Matrix *A* (shape [*C*, *r*], *r* is LoRA rank) projects token features to a low-dimensional subspace, and Matrix *B* (shape [*r*, *C*]) maps them back to the original dimension:

7 $$\begin{aligned} \text {T}&= \text {Reshape}\left( \text {X}, \left( B, H \times W, C\right) \right) \end{aligned}$$

8 $$\begin{aligned} \Delta \text {T}&= \text {T} \cdot \text {A} \cdot \sigma \text {T} \cdot \text {AC} \cdot \text {B} \cdot \alpha _r \end{aligned}$$

9 $$\begin{aligned} \text {X}'&= \text {X} + \text {Reshape}\left( \text {T} + \Delta \text {T}, \left( B, C, H, W\right) \right) \end{aligned}$$

Where:

$$\begin{aligned} \text {T}&: \text {T} \in \mathbb {R}^{B \times N \times C},\; N=H \times W=\text {number of tokens}; \\ \text {X}&: \text {feature maps}\ (\text {X}_0 \sim \text {X}_3); \\ \text {A}&: \text {A} \in \mathbb {R}^{C \times r},\; \text {projection matrix}; \\ \text {B}&: \text {B} \in \mathbb {R}^{r \times C},\; \text {reconstruction matrix}; \\ \alpha&: \alpha =16,\; \text {scaling factor to align update magnitude with original features}; \\ \sigma&: \sigma =\text {Softmax},\; \text {activation to focus on faint vessel tokens with weak signals}. \end{aligned}$$

After acquiring the LoRA-adapted features, batch normalization (BN) is applied along the channel dimension to stabilize training dynamics. Then dropout regularization is used to mitigate overfitting to spurious frequency components such as optic disc artifacts. Subsequently, a residual fusion module integrates the adapted tokens with the original aligned features. This fusion preserves the representations of head-class vessels (i.e., thick vessels) while amplifying the signals of tail-class vessels (i.e., faint vessels). Finally, a multi-layer perceptron (MLP) performs feature projection, mapping the fused tokens back to the spatial feature map format [*B*, *C*, *H*, *W*] to yield a refined frequency-domain representation with enhanced discriminability of subtle vascular structures.

### LoRA-enhanced decoder

The LoRA-enhanced decoder (denoted as DecoderLoRA) is built to upsample features while preserving tail-class (faint) vessel representation. Lightweight bilinear interpolation supports efficient upsampling with low computational cost, meeting the clinical constraint of portable deployment efficiency. Decoder-specific LoRA maintains faint vessel representation during upsampling, overcoming the long-tail challenge of “fine-branch loss during upsampling”. This integrated component design thus simultaneously resolves the long-tail vessel segmentation challenge and portable deployment requirement.

#### Lightweight upsampling with decoder LoRA integration

**Fig. 5:**
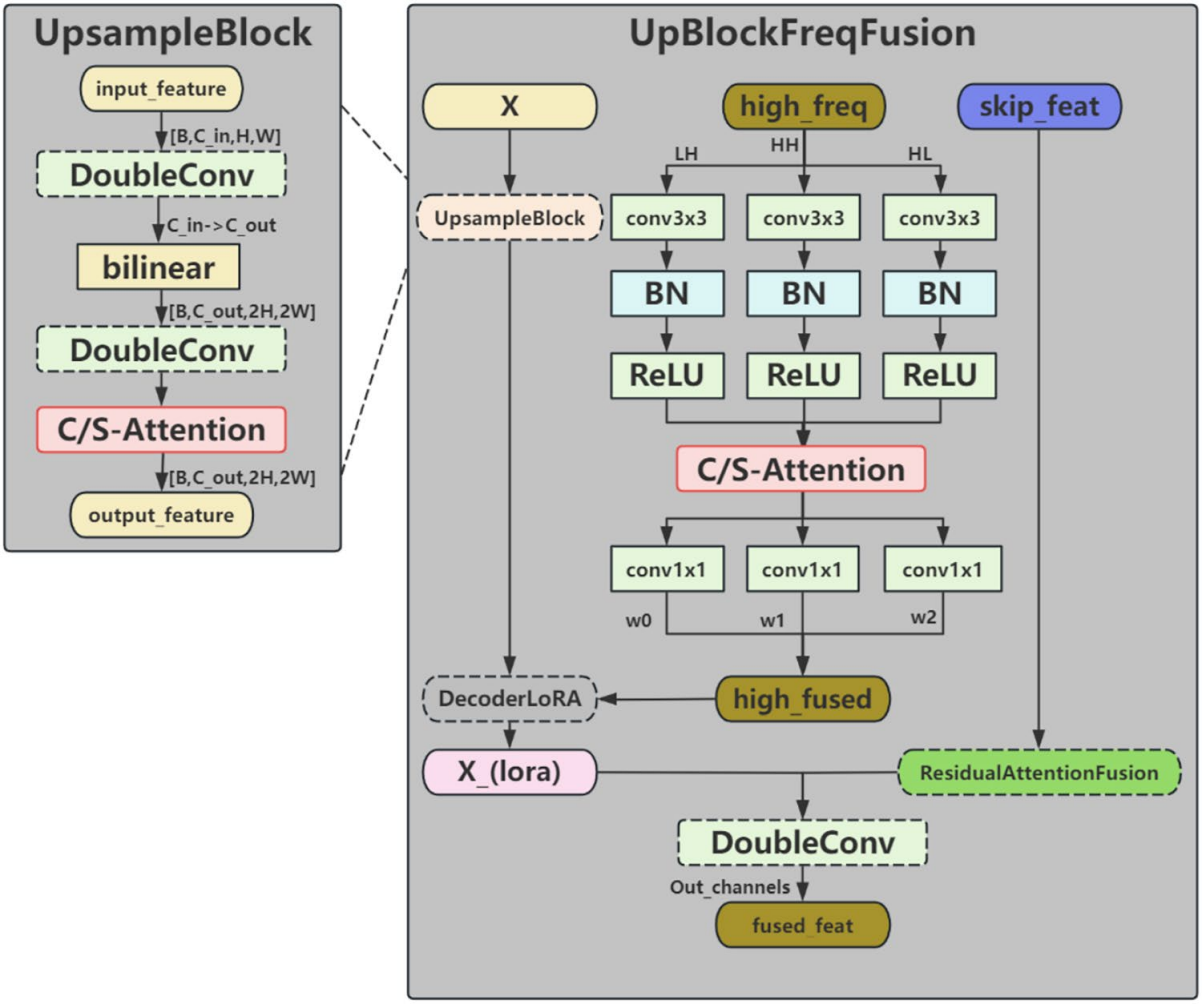
The architecture of the integrated Lightweight Upsampling-LoRA block.

Figure 5 details the unified architecture of the integrated Lightweight Upsampling-LoRA Unit. This hybrid module integrates upsampling, attention mechanisms, and low-rank adaptation techniques. It processes decoder features and high-frequency signals in sequence, with the core goal of preserving tail-class vessel representation (i.e., faint vessels). The module comprises three key components: an effective Upsample Block for lightweight upsampling, a Channel Spatial Attention module for feature weighting, and a dedicated component for tail-class enhancement.

The upsampling process within the module relies on bilinear interpolation, scaling the input feature from the preceding decoder stage $D_{i+1}$ by a factor of 2. Following this step, a DoubleConv block is used for post-processing refinement. It recovers spatial details of faint vessels lost during rescaling, ensuring $2\times$ upsampled features retain fine vascular branch information. Subsequently, the Channel Spatial Attention module dynamically assigns weights to vessel-relevant channels and spatial regions. It suppresses background artifacts such as optic disc shadows, prioritizing tail-class feature representation (i.e., faint vessels) in the final upsampled feature $D_{\text {up},i}$. The entire process can be formally expressed as:

10 $$\begin{aligned} D_{\text {up},i} = \text {C/S-Attention}\left( \text {DoubleConv}\left( \text {BilinearUpsample}\left( \text {DoubleConv}(D_{\text {up},i}), \text {scale} = 2 \right) \right) \right) \end{aligned}$$

#### High-frequency refinement and decoder LoRA adaptation

High-frequency features $\text {HF}_{\text {refined},i}$ from the Frequency-Aware Encoder (FAE) are effective at capturing faint vessel edges.However, they need task-specific adaptation to retinal topology for accurate segmentation. The UpBlockFreqFusion module meets this need by integrating high-frequency refinement with a low-rank adaptation (LoRA) unit to minimize parameter overhead. Specifically, the high_process submodule first refines high-frequency sub-bands derived from wavelet decomposition. It adopts a processing pipeline of $\text {Conv}_{3\times 3}$, BN, ReLU and C/S Attention. This pipeline enhances the representation of faint vessel edges while suppressing noise.

After that, the decoder_lora component conducts low-rank adaptation on refined high-frequency features $\text {HF}_{\text {refined},i}$, maintaining tail-class feature representation without adding redundant computations. Finally, the LoRA-adapted high-frequency features $\text {HF}_{\text {LoRA},i}$ are concatenated with $\text {D}_{\text {up},i}$ (where $\text {D}_i'$ denotes the integrated decoder feature with enhanced tail-class representation). They are then fused via a $\text {Conv}_{3\times 3}$ layer to align channel dimensions, ensuring the preservation of tail-class signals. These adaptation and fusion operations are formally expressed as:

11 $$\begin{aligned} \text {HF}_{\text {LoRA},i}&= \text {DecoderLoRA}\left( \text {HF}_{\text {refined},i}\right) \quad (r = 8/16,\; \alpha = 8) \end{aligned}$$

12 $$\begin{aligned} \text {D}_i'&= \text {Conv}_{3\times 3}\left( \text {Cat}\left( \text {D}_{\text {up},i}, \text {HF}_{\text {LoRA},i}\right) \right) \end{aligned}$$

#### Fine-branch preservation via DetailRecoveryBlock

The DetailRecoveryBlock maintains fine-branch details of faint vessels (tail-class structures) through three core steps. First, a $1\times 1$ convolution halves the channel dimension of input decoder features $D_i'$ to reduce computation and suppress background interference. Next, a stacked block with “$3\times 3$ convolution (dilation=2)+BN+ReLU” expands the receptive field, capturing cross-regional vessel branching without over-smoothing. A second $1\times 1$ convolution restores the original channel dimension for downstream compatibility.Finally, a Laplacian^35^ operator generates edge maps, which are scaled by 0.1 and fused with topology-enriched features via $1\times 1$ convolution. This balances edge enhancement and noise suppression effectively. The entire detail recovery process can be formally defined as follows: where $D_{\text {res}}$ denotes the residual topology-captured feature, and $D_i$ is the final decoder output with preserved fine branches.

13 $$\begin{aligned} D_{\text {res}}&= D_i' + \text {Conv}_{3\times 3,\,\text {dilation}=2}\left( D_i'\right) \end{aligned}$$

14 $$\begin{aligned} L&= \nabla ^2 D_{\text {res}} \quad (\nabla ^2 = \text {Laplacian kernel}) \end{aligned}$$

15 $$\begin{aligned} D_i&= D_{\text {res}} + 0.1 \cdot \text {Conv}_{1\times 1}(L) \end{aligned}$$

### Recursive Residual Attention Fusion

**Fig. 6:**
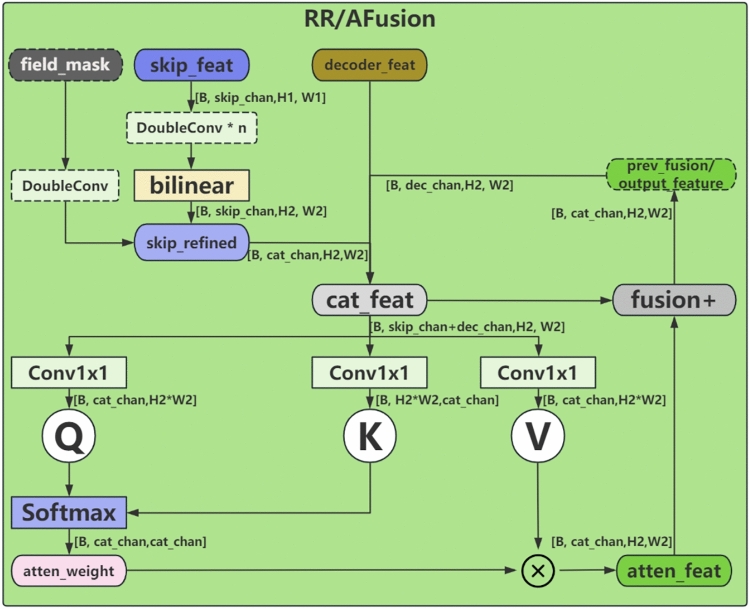
Recursive Residual Attention Fusion module.

The Recursive Residual Attention Fusion (RRAF) module overcomes the long-tail challenge of “cross-scale tail-class continuity loss”. This challenge arises when faint vessel features are overshadowed by thick vessel signals. The module utilizes a recursive loop with residual connections to preserve tail-class topological continuity. Figure 6 illustrates the structure of the RRAF module, which is designed to adaptively integrate multi-source features.

#### Core inputs and objectives

The goal is to generate a fused feature map $D_i$ that emphasizes faint vessel (tail-class) signals while preserving the structural integrity of thick vessels. At each decoder stage *i*, the RRAF module accepts four input sources.

- $D_{i+1}$(decoder_feat): The upsampled decoder feature is derived from the LoRA-enhanced decoder (e.g., $D_2$ from the up1 block at stage $i=1$). It provides high-level semantic context of vessel structures. Notably, LoRA enhancement is applied to this feature $D_{i+1}$ prior to its input into the RRAF module, refining high-frequency details of faint vessels within the decoder feature.
- $F_i$(prev_fusion): Fusion feature from the previous decoder stage (e.g., $F_2$ from stage $i = 2$ for stage $i = 3$); initialized as a zero tensor of the same dimensionality as $D_{i+1}$ when *i* corresponds to the bottom-level stage, where no prior fusion result is available.
- $S_i$(skip_feat): High-resolution feature map from the encoder (e.g., a $120\times 120$ feature map for stage $i=1$), preserving fine-grained spatial details of faint vessels such as capillary edges and branching structures.
- $\mathcal {M}_{\text {field}}$(field_mask): The binary spatial mask explicitly delineates the valid retinal tissue region in input fundus images by assigning pixel values of 1 to valid retinal areas and 0 to invalid regions. High-resolution features maintain precise spatial alignment with the original mask, allowing effective suppression of invalid regions while preserving fine details of faint vessels.

#### Key operations

The fusion process consists of three recursive steps. In attention computation, the query-key (QK) product is scaled by $\sqrt{c}$ ($c=$ channel dimension of the concatenated map). This operation highlights faint vessels and suppresses noise, completing cross-scale refinement. Notably, the bottom-level RRAF stage introduces a secondary fusion strategy: it uses the first fusion result as the new prev_fusion $F_i$ to re-refine details and reduce single-step distortion.

Step 1: Residual Feature Refinement. This step reduces noise in $S_i$ (skip_feat), the primary source of faint vessel details. It uses two stacked DoubleConv units (each: $\text {Conv}_{3\times 3}$ + Batch Normalization + ReLU). The operation enhances robustness while preserving fine spatial details and suppressing background noise.

Step 2: Valid Region Screening. First, mask alignment is performed as the foundational step: $\mathcal {M}_{\text {field}}$, with original resolution matching that of the input images, is resized via nearest-exact interpolation to align with the spatial dimensions of the refined encoder feature $S_i'$. This ensures precise spatial correspondence and boundary integrity between the mask and the feature, laying a reliable foundation for subsequent fusion operations. Subsequently, channel projection addresses the channel dimension mismatch: a $\text {Conv}_{1\times 1}$ layer projects the single-channel $\mathcal {M}_{\text {field}}$ to match the channel dimension of the refined encoder feature $S_i'$, enabling element-wise multiplication between the mask and the feature. The resulting projected mask is denoted as $\mathcal {M}_{\text {field, proj}} = \text {Conv}_{1\times 1}(\mathcal {M}_{\text {field}})$. Finally, valid region fusion is applied to suppress invalid features: element-wise multiplication between $\mathcal {M}_{\text {field, proj}}$ and the refined encoder feature $S_i'$ nullifies activations in non-retinal regions while preserving responses within valid retinal areas, yielding the refined encoder feature $S_i'' = S_i' \cdot \mathcal {M}_{\text {field, proj}}$.

Step 3: Recursive Attention Fusion (Integrate All Core Inputs). This step refines cross-scale features via single-head attention. “Recursion” is realized by transferring fusion results across decoder stages to preserve faint vessel topological continuity. First, the historical fusion result $F_i$ (prev_fusion) is projected via $\text {Conv}_{1\times 1}$ to match the channel dimension of decoder_feat $D_{i+1}$. It is weighted by an ablation-verified scaling factor (0.3) to balance feature contributions, then fused with decoder_feat $D_{i+1}$ via element-wise addition. Subsequently, the fused feature (with decoder_feat $F_i$) is concatenated along channels with the valid-region-refined feature $S_i''$ (encoder skip feature after refinement and screening). The concatenated map integrates three key cues: faint vessel spatial details $S_i''$, global semantics $D_{i+1}$, and historical experience $F_i$, then enters the single-head attention module.

### Composite loss function

We propose a Module-Synergistic Hierarchical Composite Loss Function. This loss function is tightly integrated with the core modules of LFU-NET, establishing a closed-loop mechanism centered on “feature extraction–loss optimization”. The mechanism prioritizes tail-class faint vessel learning while maintaining the segmentation accuracy of thick vessels. The composite loss integrates four synergistic components, with weights adaptively optimized via 5-fold cross-validation independently performed on each dataset. The total loss is defined as:

16 $$\begin{aligned} \mathcal {L}_{\text {total}} = \lambda _1 \cdot \mathcal {L}_{\text {FW-BF-Dice}} + \lambda _2 \cdot \mathcal {L}_{\text {LE-Edge}} + \lambda _3 \cdot \mathcal {L}_{\text {RRAF-Consist}} + \lambda _4 \cdot \mathcal {L}_{\text {LF-Suppress}} \end{aligned}$$

Where:

$\mathcal {L}_{\text {FW-BF-Dice}}$: The Frequency-Weighted Balanced Focal Dice Loss mitigates the long-tail class imbalance by leveraging dynamic frequency weights derived from the MBFC block;

$\mathcal {L}_{\text {LE-Edge}}$: The Laplacian-Enhanced Edge Loss refines the boundaries of faint vessels by utilizing the enhanced features produced by the DetailRecoveryBlock;

$\mathcal {L}_{\text {RRAF-Consist}}$: The RRAF Cross-Scale Consistency Loss ensures the preservation of topological continuity in faint vessels during the cross-scale feature fusion process within the model;

$\mathcal {L}_{\text {LF-Suppress}}$: The Low-Frequency Background Suppression Loss reduces false positives caused by background noise by utilizing the LL sub-band from the Frequency-Aware Encoder (FAE).

#### Frequency-weighted balanced focal dice loss

This loss function utilizes the MBFC block of the FAE to dynamically fuse the HH, LH, and HL sub-bands, with selective emphasis on the HL sub-band to enhance horizontal faint vessels and suppress noise through C/S-Attention. The focal term down-weights easily classified head-class samples (i.e., thick vessels), while the class balance factor ($\beta _c$) compensates for the extreme class imbalance.where $N_{\text {total}}$ is the total number of pixels, $N_c$ is the number of pixels for class *c* (background $c = 0$, vessel $c = 1$).

17 $$\begin{aligned} \beta _c = \frac{N_{\text {total}}}{2 \cdot N_c} \end{aligned}$$

In the MBFC Dynamic Frequency Weight, $\alpha _{\text {HL}}=0.6, \alpha _{\text {LH}}=0.3, \alpha _{\text {HH}} = 0.1$ (adapted to retinal vessel morphology),

$\text {HL}_{\text {norm}}$/$\text {LH}_{\text {norm}}$/$\text {HH}_{\text {norm}}$ are normalized sub-bands from FAE, and chan_weight/spat_weight are from MBFC’s C/S-Attention:

18 $$\begin{aligned} w_{\text {MBFC}}(h,w) = \alpha _{\text {HL}} \cdot \text {HL}_{\text {norm}}(h,w) \cdot \text {chan}_\text {weight} \cdot \text {spat}_\text {weight} + \alpha _{\text {LH}} \cdot \text {LH}_{\text {norm}}(h,w) + \alpha _{\text {HH}} \cdot \text {HH}_{\text {norm}}(h,w) \end{aligned}$$

The Layer-Wise Focal Term is specifically adapted to the rank configuration of the FreqTokenLoRA module to prioritize the learning of different vessel classes:

19 $$\begin{aligned} \text {Focal}(p_1) = {\left\{ \begin{array}{ll} (1 - p_1)^{2.5} & \text {deep layers (faint vessels, } r = 16\text {)} \\ (1 - p_1)^{1.5} & \text {shallow layers (thick vessels, } r = 8\text {)} \end{array}\right. } \end{aligned}$$

Final Loss:

20 $$\begin{aligned} \mathcal {L}_{\text {FW-BF-Dice}} = 1 - \frac{2 \cdot \sum _{h,w} \beta _1 \cdot w_{\text {MBFC}}(h,w) \cdot y_{\text {gt}}(h,w) \cdot p_1(h,w) \cdot \text {Focal}(p_1) + 10^{-5}}{\sum _{h,w} \beta _1 \cdot w_{\text {MBFC}}(h,w) \cdot y_{\text {gt}}(h,w) + \sum _{h,w} \beta _0 \cdot p_1(h,w) + 10^{-5}} \end{aligned}$$

#### Laplacian-enhanced edge loss

It reuses the enhanced feature $\text {after}_\text {detail}$ from the $\text {DetailRecoveryBlock}$ to compute Laplacian edges, improving the SNR of faint vessel boundaries. The $w_{\text {MBFC}}(h,w)$ further prioritizes edge loss for multi-directional faint vessels. Laplacian Edge Mask: where $\nabla ^2$ is the Laplacian operator (kernel: $$\begin{pmatrix} 0 & 1 & 0 \\ 1 & -4 & 1 \\ 0 & 1 & 0 \end{pmatrix}$$), and $(\text {after}_\text {detail})$ is the enhanced feature from $\text {DetailRecoveryBlock}$.

21 $$\begin{aligned} \textrm{E}(h,w) = {\left\{ \begin{array}{ll} 1 & |\nabla ^2 \cdot \text {after}_\text {detail}(h,w)| > 0.1 \\ 0 & \text {otherwise} \end{array}\right. } \end{aligned}$$

Final Loss: where $N_{\textrm{E}} = \sum _{h,w} \textrm{E}(h,w)$ (normalizes edge pixel imbalance).

22 $$\begin{aligned} \mathcal {L}_{\text {LE-Edge}} = -\frac{1}{N_{\textrm{E}}} \sum _{h,w} \textrm{E}(h,w) \cdot w_{\text {MBFC}}(h,w) \cdot \left[ y_{\text {gt}}(h,w) \log p_{\text {l}}(h,w) + \left( 1 - y_{\text {gt}}(h,w)\right) \log \left( 1 - p_{\text {l}}(h,w)\right) \right] \end{aligned}$$

#### RRAF cross-scale consistency loss

It leverages the $\text {prev}_\text {fusion}$ from RRAF to constrain consistency between cross-scale features, preventing topological discontinuity of faint vessels during fusion.

23 $$\begin{aligned} \mathcal {L}_{\text {RRAF-Consist}} = \frac{1}{N_{\text {vessel}}} \sum _{h,w} \text {prev}\_\text {fusion}_{\text {vessel}}(h,w) \cdot |\text {p}_{\text {l}}(h,w) - \text {p}_{\text {prev}}(h,w)| \end{aligned}$$

Where:

$$\begin{aligned} \text {prev}\_\text {fusion}_{\text {vessel}}(h,w)&: \text {Vessel mask from RRAF's previous fusion result}; \\ \text {p}_{\text {prev}}(h,w)&: \text {Predicted probability from the previous fusion stage}; \\ N_{\text {vessel}}&: N_{\text {vessel}} = \sum _{h,w} y_{\text {gt}}(h,w) ,\text {Avoids background interference}. \end{aligned}$$

#### Low-frequency background suppression loss

Uses FAE’s LL sub-band to suppress false positives from background noise (e.g., optic disc shadows), improving precision for faint vessel segmentation.

24 $$\begin{aligned} \mathcal {L}_{\text {LF-Suppress}} = \frac{1}{N_{\text {bg}}} \sum _{h,w} \text {LL}_{\text {norm}}(h,w) \cdot \left( 1 - y_{\text {gt}}(h,w)\right) \cdot \text {p}_{\text {l}}(h,w)^2 \end{aligned}$$

where:

$$\begin{aligned} \text {LL}_{\text {norm}}(h,w)&: \text {Normalized low-frequency sub-band from FAE}\end{aligned};$$

$$\left( {1 - y_{{{\mathrm{gt}}}} (h,w)} \right):{\text{Background mask}};$$

$$N_{{{\mathrm{bg}}}} :N_{{{\mathrm{bg}}}} = \sum\limits_{{h,w}} {\left( {1 - y_{{{\mathrm{gt}}}} (h,w)} \right)} \;({\text{normalizes background pixels}});$$

$${\mathrm{p}}_{{\mathrm{l}}} (h,w)^{2} :{\text{Squared penalty to enhance background suppression}}.$$

## Results

To evaluate the efficacy of the proposed LFU-Net in addressing long-tail retinal vessel segmentation, we conducted systematic experiments on four well-established retinal image datasets. The evaluation specifically focuses on three critical challenges: underrepresentation of faint vessels, ambiguity of vessel boundaries, and requirement for lightweight clinical deployability.

### Preparation and preprocessing

#### Dataset selection

LFU-Net is validated on four benchmark retinal vessel datasets covering diverse clinical scenarios.To standardize input data and enhance tail-class feature discriminability, all images are resampled to 480 $\times$ 480 pixels using bilinear interpolation.Image normalization adopts ImageNet-based statistics (mean: [0.485, 0.456, 0.406]; standard deviation: [0.229, 0.224, 0.225]). This effectively transfers generic visual representations from the pretrained MobileNetV2^26^ backbone.Furthermore, training data augmentation strategies^36^ are designed to reduce overfitting to head-class thick vessels and boost generalization performance for tail-class vessel segmentation.

DRIVE (Digital Retinal Images for Vessel Extraction)^37^: This dataset comprises 40 fundus images (32 for training, 8 for testing) with a resolution of 565 $\times$ 584 pixels. Two medical experts provided independent pixel-level annotations. It is a widely adopted benchmark for evaluating standard retinal vessel segmentation methods.

FIVES (Fundus Image Dataset for Artificial Intelligence based Vessel Segmentation)^38^: This dataset comprises 800 high-resolution fundus images (600 for training, 200 for testing) with a uniform resolution of 2048 $\times$ 2048 pixels. The dataset covers four typical fundus conditions, including diabetic retinopathy, glaucoma, age-related macular degeneration and normal cases, with 200 images per category. It serves for evaluating retinal vessel segmentation algorithms, especially for multi-disease and high-resolution clinical scenarios.

STARE (Structured Analysis of the Retina)^39^: This dataset includes 20 fundus images (10 for training, 10 for testing) at a resolution of 605 $\times$ 700 pixels. It is particularly recognized for inter-observer variability in faint vessel annotations. Thus, it serves as a testbed to assess model robustness to labeling ambiguity, an inherent challenge in tail-class structure segmentation.

CHASE DB1 (Child Heart and Health Study in England Database)^40^: This dataset contains 28 pediatric fundus images (20 for training, 8 for testing) with a resolution of 960 $\times$ 999 pixels. It features low-contrast vascular structures near the optic disc, effectively simulating the weak signal characteristics of tail-class faint vessels.

#### Training configuration and environment

AdamW^41^ is used as the optimizer to decouple the weight decay term from gradient updates, enhancing regularization for tail-class features. The detailed configuration is as follows: Differential learning rates are adopted to balance the fine-tuning of pretrained features and adaptation of task-specific modules, with distinct values assigned to different parameter groups. Specifically, the pretrained MobileNetV2 backbone and LoRA modules use a lower learning rate of $5 \times 10^{-5}$; the FAE and RRAF units adopt a higher $2 \times 10^{-4}$ to emphasize learning task-specific features more; the decoder and output layers use a moderate $1 \times 10^{-4}$ to ensure balanced upsampling and segmentation. Weight decay is set to $1 \times 10^{-5}$, applied exclusively to weight parameters (excluding biases) to reduce overfitting to head-class vessels. The momentum hyperparameters are set as $\beta _1 = 0.9$ and $\beta _2 = 0.999$. For the learning rate scheduler, Cosine Annealing with Warm Restarts (CAWR)^42^ is employed. It has an initial cycle length of 10 epochs, a cycle length multiplier of 2, and a minimum learning rate of $1 \times 10^{-7}$, preventing premature convergence. The epochs is set to 50. Early stopping is implemented with a patience of 15 epochs, monitoring the validation Dice score for faint vessels. Training is stopped if no improvement is observed over 15 consecutive epochs to avoid overfitting. The batch size is fixed at 4, balancing computational efficiency and batch diversity. The composite loss uses adaptive weights optimized via 5-fold cross-validation, taking into account dataset specificity and adjusting for domain variations (Table 4).

**Table 4:** Adaptive Loss Weights Across Datasets.

Dataset	$\boldsymbol{\lambda }_1$	$\boldsymbol{\lambda }_2$	$\boldsymbol{\lambda }_3$	$\boldsymbol{\lambda }_4$	Core rationale
DRIVE^37^	0.4	0.35	0.15	0.1	Standard vessel distribution
FIVES^38^	0.3	0.45	0.15	0.1	Multi-disease vessel distribution
STARE^39^	0.35	0.4	0.15	0.1	Ambiguous faint vessel labels
CHASE DB1^40^	0.35	0.45	0.1	0.1	Low contrast (pediatric images)

For fair assessment of LFU-Net performance and its readiness for clinical deployment, two hardware configurations are adopted for validating its training efficiency and suitability for edge deployment. An Intel Xeon W-1290 CPU (3.2 GHz), 64 GB DDR4 RAM and an NVIDIA RTX 3090 GPU (24 GB VRAM) is used for training and validating LFU-Net’s model performance. An Intel Core i7-10700 CPU (8 cores, 16 threads, 2.9GHz base frequency) is used for validating real-time inference. The inference time is measured as the average processing time per 480$\times$480 fundus image. Python 3.9 is used for code implementation. Key software dependencies include PyTorch 1.13.1 and TorchVision 0.14.1 for deep learning backbones and data utilities, OpenCV 4.7.0, Albumentations 1.3.0 and PIL 9.4.0 for image processing and data augmentation, Matplotlib 3.7.1, Seaborn 0.12.2 and TensorBoard 2.12.0 for visualization, NumPy 1.24.3 and Scikit-learn 1.2.2 for data manipulation and performance metric calculations, and TQDM 4.65.0 for displaying training progress.

#### Evaluation metrics

Dice: Measures overlap between predicted and ground-truth vessels (overall segmentation quality), defined as:

25 $$\begin{aligned} \text {Dice} = \frac{2 \times |\text {P} \cap \text {G}|}{|\text {P}| + |\text {G}| + \epsilon } \end{aligned}$$

IoU: Measures the overlap ratio between predicted and ground-truth vessel regions, complementary to Dice for comprehensive segmentation accuracy, defined as:

26 $$\begin{aligned} \text {IoU} = \frac{|\text {P} \cap \text {G}|}{|\text {P} \cup \text {G}| + \epsilon } \end{aligned}$$

Precision: Ensures minimal false positives (critical for clinical reliability), defined as:

27 $$\begin{aligned} \text {Precision} = \frac{|\text {P} \cap \text {G}|}{|\text {P}| + \epsilon } \end{aligned}$$

Sensitivity (Recall): Evaluates the ability to detect faint vessels (tail-class performance), defined as:

28 $$\begin{aligned} \text {Sensitivity} = \frac{|\text {P} \cap \text {G}|}{|\text {G}| + \epsilon } \end{aligned}$$

where $\text {P} =$ predicted vessel mask, $\text {G} =$ ground-truth mask, $\epsilon = 10^{-5}$ (avoids division by zero).

AUC-ROC: Quantifies the trade-off between true positive (TP) rate and false positive (FP) rate. It is robust to class imbalance, computed using predicted probabilities (before $\text {argmax}$) and ground-truth labels.

Faint Vessel Dice: The Dice coefficient is calculated exclusively on faint vessels with a diameter of less than 2-pixel (referred to as the tail class), serving as the primary metric for evaluating long-tail instance performance.

### Quantitative results

This section presents a comprehensive quantitative evaluation of LFU-Net in comparison with six representative baseline methods encompassing classic architectures, lightweight designs, ablation variants, and recent approaches proposed within the past three years. The baseline methods include: 1) a conventional segmentation backbone (U-Net^5^); 2) a lightweight model with a shared encoder architecture (MobileNet-U-Net); 3) an ablation variant (Abla-UNet, i.e., LFU-Net without all LoRA components) to validate the necessity of low-rank adaptation; and 4) three methods (FreqUNet^30^, MedSAGa^23^, and RCAR-UNet^43^) specifically designed for retinal vessel segmentation. Additionally, to further verify the superiority of LFU-Net in complex multi-disease clinical scenarios, we evaluate and compare it with the clinically oriented pipeline AutoMorph^44^ , and follow the same preprocessing pipeline for FIVES dataset. AutoMorph integrates image quality grading, vascular segmentation, and morphology measurement, and is trained on multiple multi-disease datasets, making it a representative benchmark for clinical application scenarios. The primary evaluation criteria correspond directly to LFU-Net’s dual design objectives: enhanced performance on faint vessel structures and suitability for clinical deployment. Experiments are conducted on four benchmark datasets to fully verify the model’s generalization ability.

#### Performance on DRIVE dataset

The DRIVE^37^ dataset contains 32 training images and 8 test images, with clear annotations and a moderate long-tail class imbalance issue.

**Table 5:** Quantitative comparison on the DRIVE.

Method	Dice	IoU	Sensitivity	Precision	AUC-ROC	Faint vessel Dice	FPS RTX 3090	FPS C-i7-10700	Params (M)
U-Net^5^	0.813	0.721	0.896	0.835	0.687	0.685	15.2	1.0	27.8
MobileNet-U-Net (Ablation)	0.801	0.668	0.705	0.889	0.829	0.673	**28.5**	**2.3**	**15.3**
Abla-UNet (Ablation)	0.831	0.710	0.765	0.909	0.875	0.712	26.2	2.1	16.1
FreqUNet^30^	0.837	0.719	0.758	0.912	0.861	0.731	10.5	0.95	29.5
MedSAGa^23^	0.842	0.727	0.769	0.915	0.878	0.738	25.6	1.9	16.8
RCAR-UNet^43^	0.851	0.741	0.775	0.921	0.893	0.752	14.6	2.2	**15.3**
LFU-Net (Ours)	**0.862**	**0.758**	**0.843**	**0.925**	**0.917**	**0.793**	27.0 ± 0.05	2.13 ± 0.03	16.4

As shown in Table 5, LFU-Net outperforms all baseline methods across key metrics, and the most notable improvements lie in tail-class-related indicators.Specifically, its Faint Vessel Dice reaches 0.793, which is a 10.6% improvement over U-Net (0.687) and 4.1–12.0% higher than recent methods like FreqUNet and MedSAGa. Such improvement stems from the design of hierarchical LoRA and FAE: LoRA amplifies sparse faint vessel features without redundancy, while FAE separates high-frequency vessel details from background noise.In terms of Sensitivity, LFU-Net achieves 0.843, which is 12.2% higher than U-Net and 6.8–13.8% superior to other baselines. This validates the effectiveness of the RRAF module, which preserves faint vessel continuity via iterative cross-scale refinement and mitigates single-step skip connection degradation.Regarding efficiency, LFU-Net maintains a lightweight design (16.4M parameters, 41% fewer than U-Net) and real-time inference (2.13 ± 0.03 FPS) despite integrating three novel components, verifying its suitability for portable clinical deployment. Furthermore, ablation studies validate each core module: MobileNet-U-Net (without LoRA and FAE) shows an additional 3.9% drop in Faint Vessel Dice compared to Abla-UNet, highlighting FAE’s role in refining subtle vessel features. Abla-UNet (LFU-Net without LoRA) exhibits an 8.1% reduction in Faint Vessel Dice, confirming the essential role of hierarchical LoRA.

#### Performance on STARE dataset

The STARE^39^ dataset (10 training/10 testing images) is characterized by inter-observer annotation ambiguity and uneven illumination, making it a critical test for the robustness of tail-class segmentation under label uncertainty (Table 6).

**Table 6:** Quantitative comparison on the STARE.

Method	Dice	IoU	Sensitivity	Precision	AUC-ROC	Faint vessel dice	FPS RTX 3090	FPS C-i7-10700	Params (M)
U-Net^5^	0.821	0.697	0.732	0.898	0.842	0.695	14.8	1.0	27.8
MobileNet-U-Net (Ablation)	0.812	0.683	0.718	0.893	0.836	0.681	**27.9**	**2.3**	**15.3**
Abla-UNet (Ablation)	0.843	0.717	0.785	0.917	0.892	0.735	26.2	2.1	16.1
FreqUNet^30^	0.853	0.730	0.785	0.920	0.891	0.752	10.2	0.95	29.5
MedSAGa^23^	0.848	0.723	0.779	0.918	0.887	0.746	24.8	1.9	16.8
RCAR-UNet^43^	0.859	0.737	0.792	0.924	0.901	0.768	13.9	2.2	**15.3**
LFU-Net (Ours)	**0.875**	**0.754**	**0.851**	**0.932**	**0.923**	**0.804/0.806**	27.0 ± 0.05	2.13 ± 0.03	16.4

LFU-Net achieves the highest average Dice (0.875), Sensitivity (0.851), and Faint Vessel Dice (0.802/0.806) across datasets. This demonstrates strong adaptability to diverse conditions, including annotation variability and low-contrast imaging.

#### Performance on CHASE DB1 dataset

CHASE DB1^40^ contains 20 training and 8 testing images, consisting of low-contrast pediatric fundus images. Faint vessels here are easily obscured by optic disc shadows and uneven illumination. This dataset evaluates LFU-Net’s ability to detect weak tail-class signals under poor imaging conditions (Table 7).

**Table 7:** Quantitative comparison on the CHASE DB1.

Method	Dice	IoU	Sensitivity	Precision	AUC-ROC	Faint vessel dice	FPS RTX 3090	FPS C-i7-10700	Params (M)
U-Net^5^	0.805	0.674	0.713	0.892	0.831	0.681	14.5	1.0	27.8
MobileNet-U-Net (Ablation)	0.792	0.656	0.698	0.885	0.822	0.665	**27.8**	**2.3**	**15.3**
Abla-UNet (Ablation)	0.825	0.702	0.753	0.906	0.870	0.715	24.3	2.1	16.1
FreqUNet^30^	0.829	0.708	0.745	0.908	0.863	0.725	9.8	0.95	29.5
MedSAGa^23^	0.834	0.715	0.758	0.913	0.875	0.732	23.7	1.9	16.8
RCAR-UNet^43^	0.842	0.726	0.761	0.918	0.886	0.741	13.9	2.2	**15.3**
LFU-Net (Ours)	**0.856**	**0.748**	**0.824**	**0.927**	**0.905**	**0.782**	25.9 ± 0.05	2.13 ± 0.03	16.4

The CHASE DB1^40^ dataset demonstrates LFU-Net’s superior capability in enhancing weak tail-class signals.Specifically, it achieves a Sensitivity of 0.824, a 11.1% improvement over U-Net and a 6.3–12.6% gain compared to other baseline methods.In addition, LFU-Net reaches a Faint Vessel Dice of 0.782, surpassing RCAR-UNet (0.741) by 4.1%. This confirms the RRAF module effectively preserves fine vessel structures lost in conventional single-step upsampling. Notably, the model maintains high computational efficiency at 2.13 ± 0.03 FPS, meeting real-time clinical deployment requirements even under low-contrast imaging conditions.

#### Performance on FIVES dataset

To verify generalization, we conduct training and evaluation on the FIVES^38^ dataset. The training set consists of 600 images, which cover diabetic retinopathy, glaucoma, age-related macular degeneration, and normal fundus cases. Evaluation is performed on the independent 200-image test set.

**Table 8:** Quantitative comparison on the FIVES.

<thead>Method	Dice	IoU	Sensitivity	Precision	AUC-ROC	Faint vessel dice	FPS RTX 3090	FPS C-i7-10700	Params (M)
U-Net^5^	0.791	0.655	0.710	0.889	0.829	0.678	14.3	1.0	27.8
MobileNet-U-Net (Ablation)	0.779	0.639	0.692	0.881	0.818	0.662	**27.2**	**2.3**	**15.3**
Abla-UNet (Ablation)	0.816	0.690	0.748	0.903	0.867	0.708	23.8	2.1	16.1
FreqUNet^30^	0.823	0.699	0.740	0.906	0.860	0.719	9.5	0.95	29.5
MedSAGa^23^	0.829	0.707	0.753	0.910	0.872	0.727	23.1	1.9	16.8
RCAR-UNet^43^	0.837	0.718	0.757	0.915	0.883	0.736	13.5	2.2	**15.3**
AutoMorph^44^	0.785	0.645	0.805	0.770	0.878	0.670	1.5	0.8	31.2
LFU-Net (Ours)	**0.850**	**0.739**	**0.819**	**0.923**	**0.902**	**0.775**	25.3 ± 0.05	2.13 ± 0.03	16.4

As shown in Table 8, the FIVES-trained LFU-Net achieves a Dice score of 0.850, a IoU of 0.739, a Sensitivity of 0.819, and a Faint Vessel Dice score of 0.775. Compared with the corresponding models trained on the other three benchmark datasets (DRIVE: 0.862 Dice, STARE: 0.875 Dice, CHASE DB1: 0.856 Dice), our model still maintains competitive performance. Compared with AutoMorph, LFU-Net achieves significant advantages in core segmentation metrics: Dice (0.850 vs. 0.780, +7.0%), IoU (0.739 vs. 0.640, +9.9%), and Faint Vessel Dice (0.775 vs. 0.665, +11.0%). Notably, LFU-Net yields the highest Faint Vessel Dice score on FIVES among all comparison methods, which fully demonstrates the superior generalization capability of the proposed model. This confirms that LFU-Net’s frequency-domain enhancement and hierarchical LoRA design are more effective in capturing faint vessels in multi-disease scenarios. In terms of deployment efficiency, LFU-Net has 34% fewer parameters (16.4M vs. 31.2M) and 2.7 times faster CPU inference speed (2.13 ± 0.03 FPS vs. 0.8 FPS), which is more suitable for portable clinical devices.

### Qualitative results

To complement the quantitative analysis, this section presents qualitative evaluations of LFU-Net and baseline methods on four benchmark datasets. All visualizations follow a consistent layout: each subfigure displays the input fundus image, the corresponding ground truth (GT) vessel mask.All segmentation results are visualized at the original input resolution to maintain spatial fidelity.

#### Performance on DRIVE dataset

**Fig. 7:**
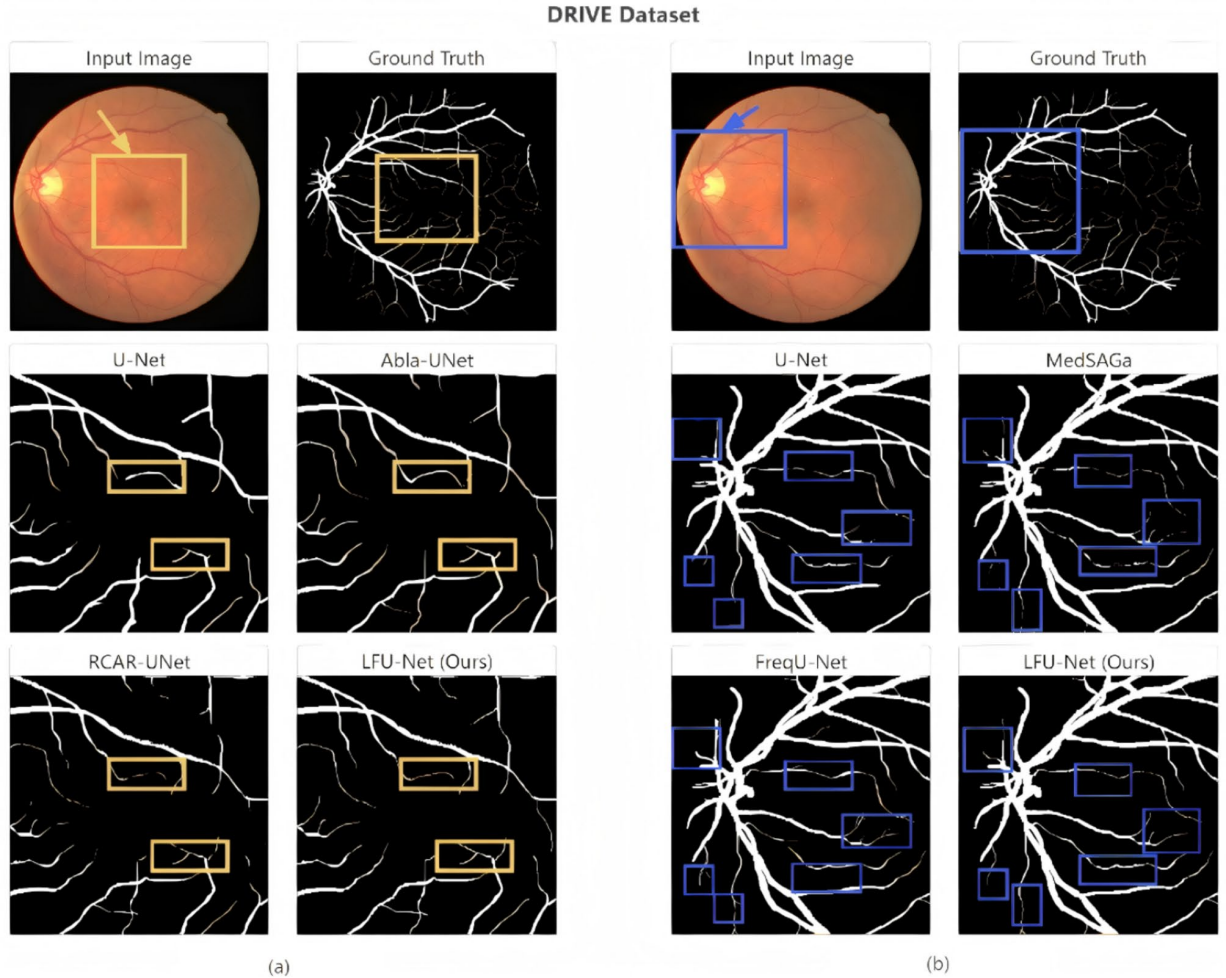
Qualitative results on DRIVE dataset.

Figure 7 shows segmentation results on the DRIVE^37^ dataset, which has clear vessel annotations and moderate long-tail imbalance. Its key challenges are preserving the continuity of faint capillary branches (yellow box) and suppressing false positives near the optic disc (blue box).

Among the baseline methods, U-Net^5^ fails to accurately segment capillary branches with diameters less than 2-pixel, as its single-step skip connection fusion mechanism tends to lose fine-grained features of tail-class vessels during the upsampling process and lacks targeted enhancement for sparse and faint vessel signals. Abla-UNet (Ablation) mitigates partial background noise through the FAE module but still overlooks a substantial proportion of faint vessels due to insufficient amplification of tail-class features, thereby underscoring the necessity of LoRA’s fixed rank allocation. FreqUNet^30^ captures certain high-frequency vessel details; however, it introduces noticeable false positives near the optic disc, a limitation attributed to its fixed frequency-band selection that cannot effectively differentiate between faint vessels structures and noise. MedSAGa^23^ employs gradient-based low-rank projection to improve memory efficiency but utilizes a non-hierarchical low-rank architecture, which limits its ability to preserve topological continuity in faint capillaries–resulting in fragmented segmentation at branching regions due to inadequate adaptation to the sparsity of retinal vasculature. RCAR-UNet^43^ incorporates a retinal-specific coarse attention mechanism designed to prioritize thick vessel segmentation, yet it performs poorly on faint capillaries; its attention maps fail to adequately focus on vessels with diameters under 2-pixel, leading to discontinuous segmentation outcomes.

#### Performance on STARE dataset

**Fig. 8:**
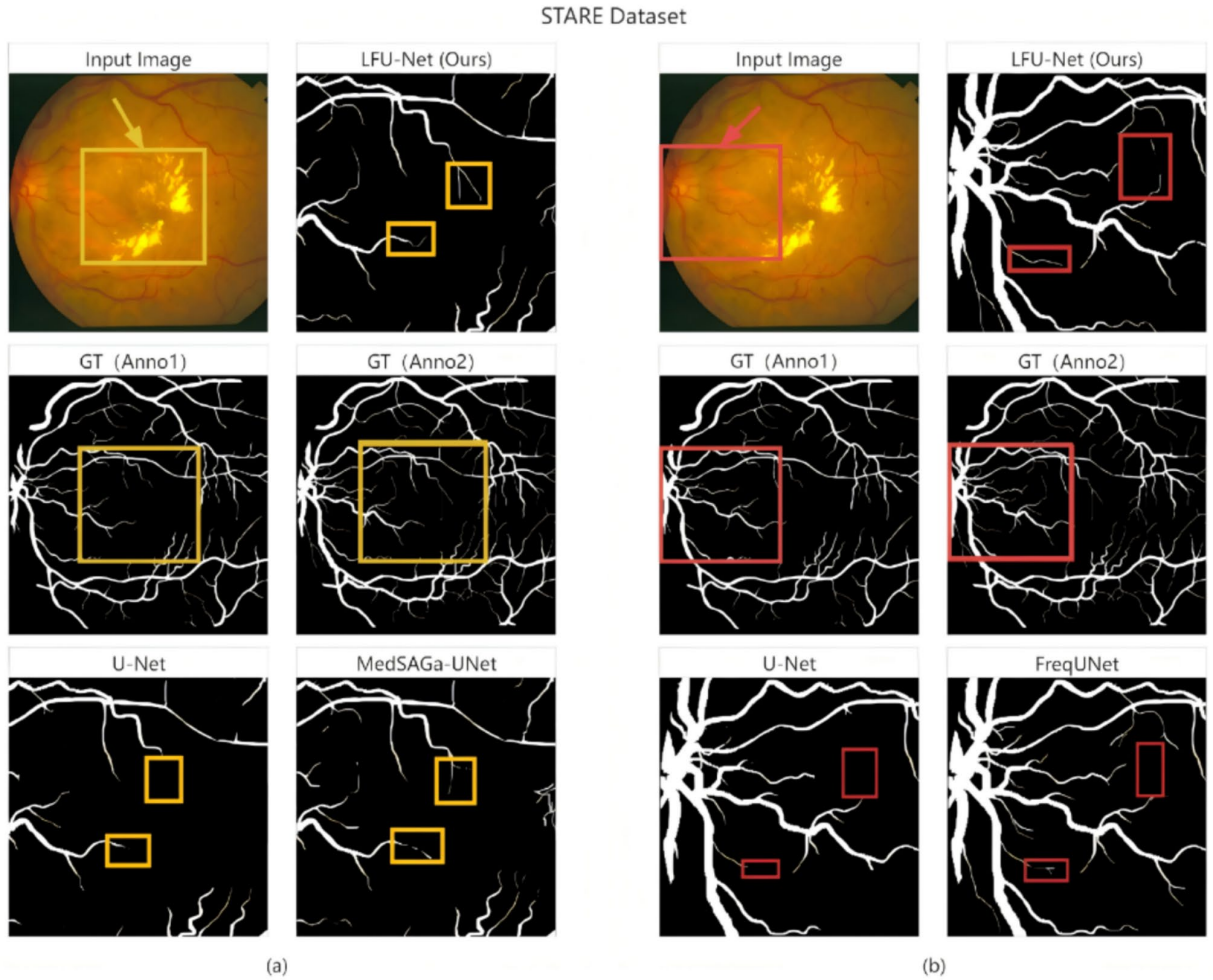
Qualitative results on STARE dataset.

Figure 8 shows segmentation results on the STARE^39^ dataset. It features notable inter-observer annotation ambiguity for faint vessels (vessels< 2-pixel), serving as a critical test for model robustness against label noise.

MedSAGa^23^ produce fragmented segmentation for ambiguously annotated vessels (yellow box), as they employs gradient-based low-rank projection that prioritizes thick vessels. FreqUNet^30^ over-segments ambiguous regions (red box) due to fixed frequency bands that cannot adapt to label uncertainty.

#### Performance on CHASE DB1 dataset

**Fig. 9:**
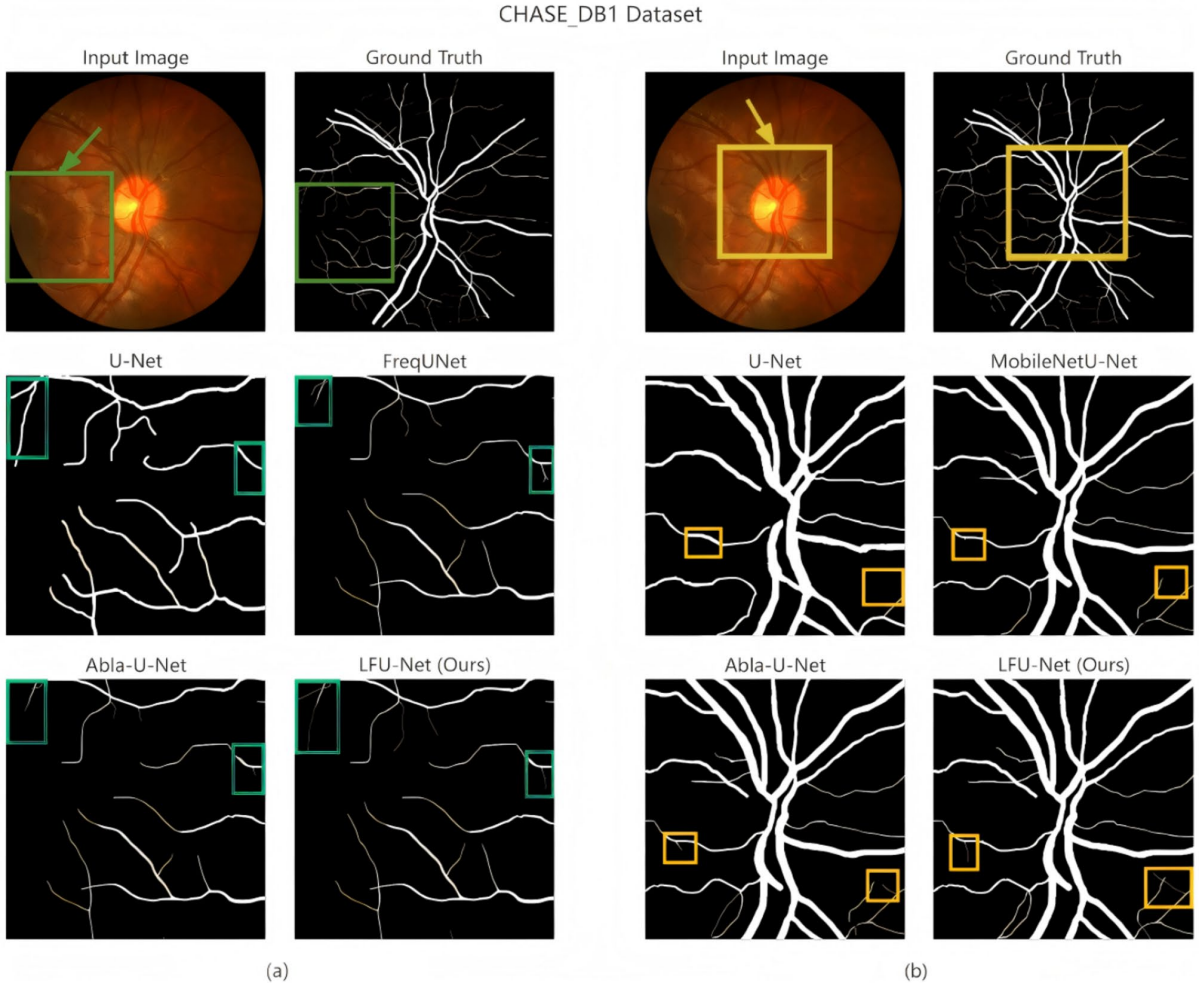
Qualitative results on CHASE DB1 dataset.

Figure 9 shows segmentation results on CHASE DB1^40^, which consists of low-contrast pediatric fundus images. Faint vessels here are easily obscured by optic disc shadows and uneven illumination, testing LFU-Net’s ability to amplify weak tail-class signals.

U-Net and MobileNet-U-Net misclassify low-contrast capillary branches as background, as their spatial convolutions fail to amplify weak intensity differences. Abla-UNet (without FAE) shows slight improvement but still misses vessels in shadowed regions, as it lacks frequency-domain noise suppression.LFU-Net clearly segments faint vessels in low-contrast regions (green box) and suppresses optic disc shadows (yellow box). This is driven by FAE’s Daubechies DWT decomposition, which separates high-frequency vessel details from low-frequency background noise. The MBFC block further weights high-frequency sub-bands to enhance faint vessel signals.

#### Performance on FIVES dataset

**Fig. 10:**
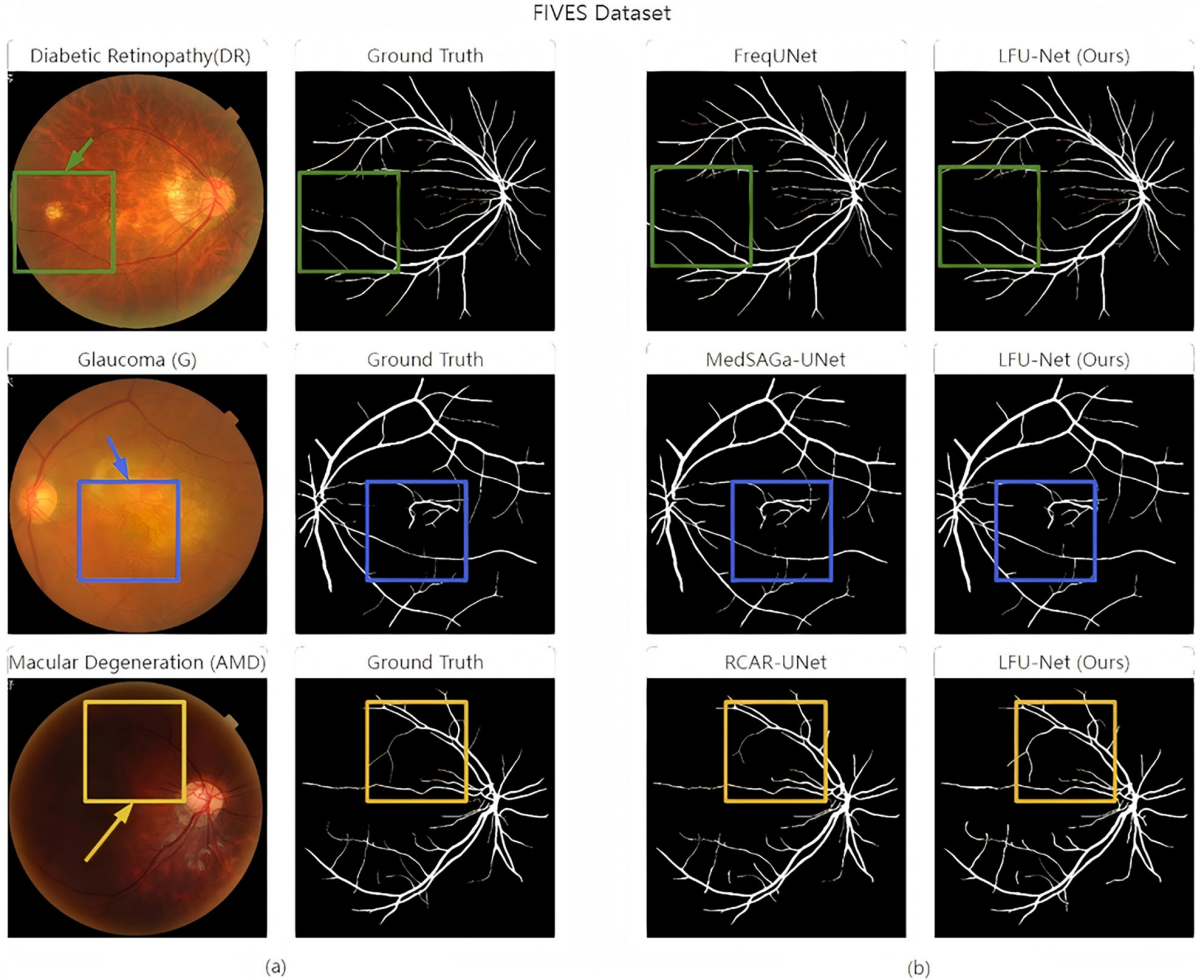
Qualitative results on FIVES dataset.

Figure 10 shows qualitative results on the FIVES^38^ dataset, focusing on two key challenges: segmenting faint vessels in DR pathological regions (green box) and suppressing false positives near glaucoma-related optic disc changes (blue box). Compared with baseline methods, LFU-Net effectively preserves the continuity of capillary branches and avoids misclassifying pathological exudates as vessels. This advantage stems from the FAE module’s ability to separate high-frequency vessel details from pathological artifacts, and the RRAF module’s recursive fusion that prioritizes tail-class features. Even on unseen imaging styles (e.g., AMD fundus images with drusen by yellow box), LFU-Net maintains accurate segmentation, confirming its strong generalization.

#### Summary of qualitative findings

Qualitative analysis confirms three core advantages of LFU-Net that align with clinical requirements for retinal vessel segmentation.First, it delivers superior tail-class faint vessel segmentation, reliably capturing capillary branches with diameters less than 2-pixel that are often overlooked by baselines like U-Net. This capability stems from the synergy of Hierarchical LoRA (hierarchical fixed ranks: r=8 for shallow layers, r=16 for deep layers) that enhances sparse vessel features, and the MBFC block within FAE that effectively separates high-frequency vessel edges from background noise.Second, LFU-Net achieves effective suppression of false positives in clinically ambiguous regions, such as optic disc shadows and pathological exudates, thereby serving as a critical safeguard against diagnostic errors. In cases like STARE^39^, where faint vessels exhibit ambiguous annotations, LFU-Net avoids over-segmentation and misclassification. This is achieved through the combined use of FAE’s adaptive frequency weighting and the Low-Frequency Background Suppression Loss, thereby ensuring segmentation precision closely aligned with expert reference standards.Third, it maintains vascular topological continuity by mitigating the fragmentation of faint vessels commonly observed in U-Net’s single-step skip fusion architecture. The RRAF module enables recursive cross-scale refinement, reconstructing continuous vessel structures even in occluded or low-contrast regions, outperforming baseline models that produce discontinuous segmentation gaps.

Notably, the FIVES^38^ dataset results further confirm LFU-Net’s robustness and generalization. Specifically, compared with the clinically oriented pipeline AutoMorph, LFU-Net shows distinct qualitative advantages: AutoMorph tends to miss faint capillary branches in diabetic retinopathy (DR) pathological regions and generate false positives near glaucoma optic discs due to insufficient suppression of pathological artifacts, which is consistent with its lower Faint Vessel Dice of 0.670. In contrast, LFU-Net effectively preserves vascular continuity and suppresses background interference, which visually echoes its quantitative lead. This adaptability to diverse pathological changes and imaging styles validates that LFU-Net learns universal retinal vessel features. Collectively, these results demonstrate that LFU-Net’s key components work synergistically to address the triple core challenge of long-tail retinal vessel segmentation: the underrepresentation of faint vessels, noise-induced boundary ambiguity, and excessive parameters that hinder portable deployment.

### Ablation studies

To examine the contribution of LFU-Net’s core components, targeted ablation experiments are carried out on the DRIVE dataset with consistent training configurations across all model variants. The evaluation focuses on long-tail segmentation-related metrics (Faint Vessel Dice, Sensitivity) and overall performance indicators (Dice, AUC-ROC).For Hierarchical FreqTokenLoRA, its necessity is evaluated by comparing with Abla-UNet (baseline without all LoRA components) and variants with fixed rank allocations (r=4, 8, 16, 32 for all layers), validating the rationality of the hierarchical rank design.Regarding the FAE, two ablation variants are designed: one omits the entire FAE module to investigate the role of frequency-domain decomposition; the other removes C/S-Attention in MBFC to evaluate the attention mechanism’s ability to distinguish vessel structures from background noise.For the Recursive Residual Attention Fusion (RRAF) module, a dedicated ablation variant replaces it with conventional skip connections and simple feature concatenation, verifying its role in preserving the topological continuity of faint vessels during cross-scale fusion.For the composite loss function, it is replaced with the standard Dice Loss (excluding frequency weighting and edge-aware regularization) to assess its efficacy in mitigating long-tail class imbalance.

**Table 9:** Quantitative results of ablation experiments on DRIVE dataset.

Method	Dice	Sensitivity	Precision	AUC-ROC	Faint vessel dice	Parans(M)
LFU-Net (Ours)	**0.862**	**0.843**	**0.925**	**0.917**	**0.793**	16.4
LFU-Net (w/o RRAF)	0.851	0.800	0.924	0.899	0.751	16.2
Abla-UNet	0.831	0.765	0.909	0.875	0.712	16.1
LFU-Net (fixed-r=4)	0.828	0.775	0.908	0.881	0.725	**15.8**
LFU-Net (fixed-r=8)	0.841	0.802	0.918	0.901	0.756	16.1
LFU-Net (fixed-r=16)	0.845	0.810	0.919	0.905	0.763	16.7
LFU-Net (fixed-r=32)	0.839	0.801	0.915	0.898	0.752	18.9
LFU-Net (w/o FAE)	0.825	0.771	0.907	0.883	0.721	15.9
LFU-Net (FAE w/o C/S-Attention)	0.838	0.795	0.916	0.897	0.748	16.0
LFU-Net (w/ Standard Dice)	0.835	0.789	0.912	0.892	0.733	16.4

Ablation experiment results demonstrate the necessity of LFU-Net’s core components in enhancing long-tail segmentation performance.Hierarchical FreqTokenLoRA boosts tail-class features across different levels and layers. Removing all LoRA components (replacing with Abla-UNet) leads to an 8.1% drop in Faint Vessel Dice. To validate the sensitivity of LoRA rank selection and the optimality of our hierarchical fixed-rank design, we conducted supplementary experiments with uniform fixed ranks (r = 4, 8, 16, 32) on the DRIVE dataset. As shown in Table 9, Fixed-r = 4 (Dice = 0.828; Faint Vessel Dice = 0.725) suffers from insufficient modeling capacity for sparse, faint vessels in deep layers, leading to the lowest overall and tail-class performance. Fixed-r = 8 (Dice = 0.841; Faint Vessel Dice = 0.756) and fixed-r = 16 (Dice = 0.845; Faint Vessel Dice = 0.763) achieve optimal performance for shallow and deep layers, respectively, as their ranks match the feature sparsity at each layer. Fixed-r = 32 (Dice = 0.839; Faint Vessel Dice = 0.752) introduces redundant parameters (18.9M vs. 16.7M for r = 16) and mild overfitting, resulting in a slight drop in all key metrics. Notably, its Faint Vessel Dice is 0.011 lower than that of r = 16, confirming that excessive rank does not enhance tail-class representation but increases computational cost. Our hierarchical design integrates the advantages of r = 8 and r = 16, achieving a Dice of 0.862 and a Faint Vessel Dice of 0.793, surpassing all uniform fixed-rank configurations. This confirms that rank selection must be tailored to the hierarchical feature sparsity of retinal vessels and validates the necessity of our proposed hierarchical LoRA design. The Frequency-Aware Encoder (FAE) filters high-frequency vessel information and separates it from noise. Ablating the entire FAE results in a 7.2% decline in Faint Vessel Dice, and removing C/S-Attention causes a 4.5% reduction in the same metric.The Recursive Residual Attention Fusion (RRAF) module plays an essential role in preserving cross-scale vascular topological continuity. Replacing it with regular skip connections or simple feature concatenation leads to a 4.2% drop in Faint Vessel Dice.The Module-Synergistic Composite Loss makes better use of tail-class samples and targets faint vessel structure recovery. Models using this loss outperform those with standard Dice Loss, achieving a 6.0% improvement in Faint Vessel Dice.Notably, these modules form a synergistic loop: FAE decouples high-frequency tail-class features from noise, Hierarchical FreqTokenLoRA efficiently amplifies sparse signals, RRAF maintains topological continuity during cross-scale fusion, and the composite loss optimizes the model toward faint vessel recovery. This integration enables LFU-Net to achieve superior long-tail segmentation performance while retaining a lightweight architecture, laying a solid foundation for clinical deployment in portable ophthalmic devices.

## Conclusions

This study introduces LFU-Net, a lightweight and clinically deployable framework for long-tail retinal vessel segmentation. It helps mitigate three key limitations of existing methods: faint vessel underrepresentation, noise-induced boundary ambiguity, and parameter redundancy hindering portable deployment. By coupling frequency-domain analysis, hierarchical low-rank adaptation, and recursive attention fusion, LFU-Net achieves robust performance across four benchmark datasets (DRIVE, STARE, CHASE DB1, FIVES). It achieves Faint Vessel Dice scores of 0.793, 0.806, 0.782 and 0.775 respectively, representing 4.1–12.0% improvements over recent models. The model attains a Sensitivity of 0.843 on DRIVE and 0.819 on FIVES, thus effectively picking out sparse capillary structures vital to early disease detection. The Frequency-Aware Encoder (FAE) equipped with the Multi-Branch Frequency Convolution (MBFC) block isolates high-frequency vessel details from background noise through Daubechies wavelet decomposition and channel-spatial attention.Meanwhile, Hierarchical FreqTokenLoRA dynamically adapts to feature sparsity with rank configurations (r=8 for shallow thick vessels, r=16 for deep faint vessels), enabling efficient adaptive enhancement of tail-class features. Complemented by the Recursive Residual Attention Fusion (RRAF) module, which preserves vascular topological continuity through iterative cross-scale feature integration, LFU-Net maintains a compact architecture with 16.4 million parameters. This is 41% fewer than U-Net (27.8M), supporting real-time inference at 2.13 ± 0.03 FPS on an Intel Core i7-10700 and making it well-suited for portable ophthalmic devices. Compared with the clinically oriented pipeline AutoMorph, LFU-Net achieves 7.0% higher Dice and 11.0% higher Faint Vessel Dice on the multi-disease FIVES dataset, while having 47.4% fewer parameters and 2.7 times faster CPU inference speed, demonstrating superior performance and clinical applicability. Ablation studies confirm the indispensable contribution of each core component, with Faint Vessel Dice dropping by 8.1% and 7.2% upon removing LoRA and FAE respectively on DRIVE. While the model has these merits, it still has certain limitations, such as its reliance on manually designed hierarchical fixed LoRA ranks (i.e., r=8 for shallow layers, r=16 for deep layers) and suboptimal performance on datasets with significant annotation ambiguity. We plan to focus future efforts on developing self-adaptive rank allocation strategies that can dynamically adjust ranks while avoiding over-adaptation to noise, and expanding the framework for multi-class retinal segmentation with semi-supervised learning , to enhance clinical applicability and generalization.

## Data Availability

The public datasets utilized in this study (DRIVE, STARE, FIVES and CHASE db1) are publicly available as standard benchmark datasets in the field. The analysis data generated in this work have been presented in Tables (4, 5, 6, 7 and 8) of the manuscript.
